# Code-Switching Does Not Equal Code-Switching. An Event-Related Potentials Study on Switching From L2 German to L1 Russian at Prepositions and Nouns

**DOI:** 10.3389/fpsyg.2020.01387

**Published:** 2020-06-23

**Authors:** Jan Patrick Zeller

**Affiliations:** ^1^Speech and Music Lab, Carl von Ossietzky University Oldenburg, Oldenburg, Germany; ^2^Faculty of Humanities, Institute for Slavistics, University of Hamburg, Hamburg, Germany

**Keywords:** code-switching, word class, event-related potentials, N400, late positive complex, phonological mismatch negativity

## Abstract

Studies on event-related potentials (ERP) in code-switching (CS) have concentrated on single-word insertions, usually nouns. However, CS ranges from inserting single words into the main language of discourse to alternating languages for larger segments of a discourse, and can occur at various syntactic positions and with various word classes. This ERP study examined native speakers of Russian who had learned German as a second language; they were asked to listen to sentences with CS from their second language, German, to their first language, Russian. CS included either a whole prepositional phrase or only the lexical head noun of a prepositional phrase. CS at nouns resulted in a late positive complex (LPC), whereas CS at prepositions resulted in a broad early negativity, which was followed by an anterior negativity with a posterior positivity. Only in the last time window (800–1000 ms) did CS at prepositions result in a broad positivity similar to CS at nouns. The differences between both types of CS indicate that they relate to different psycholinguistic processes.

## Introduction

Code-switching (CS) is “the use of several languages or dialects in the same conversation or sentence by bilingual people. It affects practically everyone who is in contact with more than one language or dialect, to a greater or lesser extent” (Gardner-Chloros, 2009, p. 4). Because CS promises to provide a window into the organization and control of the languages in the bilingual mind, it has been considered as “the central issue in bilingualism research” (Milroy and Muysken, 1995, p. 7). Hence, it is not surprising that CS has received attention from neuro/psycholinguistics. Several studies have examined the processing of CS, with some focusing specifically on the brain’s response to CS, as measured with EEG (for overviews cf. Kutas et al., 2009; Van Hell et al., 2015, 2018). Most of these studies have considered the processing of the CS of single nouns (among others, Moreno et al., 2002; Liao and Chan, 2016; Ruigendijk et al., 2016) or other meaningful lexical elements (on verbs, see Ng et al., 2014; on adjectives, see van der Meij et al., 2011) as one instantiation of CS. Most studies have found some type of early negativity (e.g., an N400, see below for details) and/or a late positive complex (LPC, see below for details) for processing CS, which sometimes depend on the direction of the switch, such as from the first language (L1) into the second (L2), or vice versa, or on the proficiency of the L2. Researchers have argued that these components are indicators of problems with processing lexical-semantic information (for the N400, see Kutas and Federmeier, 2000, for an overview), as well as with syntactic and/or general processing costs (for the LPC, see Van Petten and Luka, 2012).

In natural speech, however, CS does not occur only with single nouns, verbs, or adjectives. It can involve different word classes, ranging from inserting single words into the main language of discourse to alternating languages for larger segments of discourse, and can happen at various positions in the sentence. This does not mean that all these instances of CS can be lumped together or that certain aspects, such as word class or syntactic structure, are extraneous to CS. On the contrary, CS in corpora of natural speech has often been studied with respect to structurally different types of CS, structural restrictions (i.e., when and where CS is likely to occur), or the likelihood of different word classes being switched (see Pfaff, 1979; Sankoff and Poplack, 1981; Di Sciullo et al., 1986; Myers-Scotton, 1993). Some contact linguists have assumed that different CS phenomena differ psycholinguistically (e.g., Muysken, 2000, p. 3), while others have explicitly challenged psycholinguists to address these issues (e.g., Myers-Scotton, 2006). So far, the internal differentiation of CS has not received much attention from psycholinguists. For example, to my knowledge, only two ERP studies have addressed the switching of larger parts of a sentence (Litcofsky and Van Hell, 2017; Fernandez et al., 2019), and no ERP study has compared different CS types directly. Moreover, only one study has examined the effect of word class on the processing of CS (Ng et al., 2014), and none has studied CS in word classes other than nouns, verbs, or adjectives.

### Code-Switching in Prepositional Phrases

The following examples represent two common types of CS:

(1)
  a. Der Kapitän steuert das Schiff in diesen *port*.
    The captain steers the ship into this_German_ harbor_Russian_.
  b. Der Kapitän steuert das Schiff *v ėtot port*.
    The captain steers the ship_German_ into this harbor_Russian_.

In (1a), CS takes place at the noun *port* “harbor,” that is, a Russian noun that is part of a PP with a German preposition and determiner. In (1b), the CS occurs at the Russian preposition *v* “in,” and the whole PP is in Russian. There are hints that CS at nouns (1a) is processed differently from CS at prepositions (1b). First, it seems reasonable to suppose that prepositions and nouns are processed differently in monolingual language. However, because the same preposition can fulfill different functions, the picture is rather complex. Prepositions can be categorized as functional or lexical elements (cf. Corver and van Riemsdijk, 2001). As an analogy to classifications of case, other authors have argued for a three- or even four-way distinction (Hentschel, 2003)^1^. One might suspect that prepositions are processed differently depending on the function in which they are used. This was studied by Chanturidze et al. (2019). Violations of lexical prepositions (locative prepositions as in *to be on the table*) elicited an N400, and violations of functional (subcategorized) prepositions (as in *waiting for*) a P600. Differences were also found for the noun of the PP: the authors reported a P600 for nouns with violations of both types of prepositions, but an N400 was found only with lexical prepositions. Violations of lexical prepositions, but not of subcategorized prepositions, also elicited an N200 effect [a phonological mismatch negativity (PMN)]. Given these clear differences in monolingual speech, it seems plausible that the processing of switched prepositions depends on these differences. In the examples above, the prepositions contribute to the semantics of the clause as directional prepositions and can hence be classified as lexical elements.

Moreover, nouns are open-class items, whereas prepositions are closed-class items. Brown et al. (1999) showed that closed-class words elicit qualitatively different ERPs compared with open-class words, something that could not be explained by word length or frequency effects. They found a similar early negativity for both word classes and a typical N400 pattern for open-class words, but closed-class words elicited a slow frontal negativity (350–500 ms) that they related to the contingent negative variation (CNV; Hillyard, 1973). The CNV has been argued to reflect the processing of a closed-class word, as a syntactic signal that a new head (Van Petten and Kutas, 1991) or, a little less specifically, a meaningful word is coming up (Brown et al., 1999).

Second, although it is difficult to say whether CS at nouns or at prepositions is more “natural,” it is clear from corpus linguistic studies that prepositions and nouns behave differently with respect to CS. Single nouns are the most frequently switched elements (see Matras, 2009, p. 133f.), and the literature agrees that single prepositions are rarely switched (Pfaff, 1979; Bentahila and Davies, 1983; Joshi, 1984; Muysken, 2000, pp. 232–239). However, when it comes to whole PPs, not single prepositions, the switching probability increases. Backus (1996) reported a hierarchy of “switchability” in Turkish-Dutch CS, as follows: nouns > verbs > adverbs > adjectives > PPs > conjunctions > pronouns. For (Judeo-)Spanish-Hebrew CS, Berk-Seligson (1986) identified the following hierarchy: nouns > adverbs > adjectives > conjunctions > verb phrases > PPs > pronouns > interrogatives > verbs. Hence, in both hierarchies, PPs are less likely to be switched than single nouns, but word classes and syntactic units are mixed in these hierarchies. For the present study, the relevant comparison is between CS of whole PPs and CS of nouns *within* PPs. The question is whether it is more likely to switch a whole PP or only the noun in a PP, but here the picture becomes less clear. Bentahila and Davies (1983) found many instances of switching whole PPs in Arabic-French CS, as did Clyne (1987) in German-English CS. It has even been argued that it is impossible to combine a preposition in one language with a noun in another language (Lipski, 1977, on English-Spanish). In the corpus study by Pfaff (1979), however, English nouns within a Spanish PP were found to be much more frequent than switches of the whole PP and also more frequent than an English article and noun after a Spanish preposition (for similar results, see Sankoff and Poplack, 1981).

The question about CS with regard to PPs relates to a more general question about CS with regard to DPs. Numerous studies have examined which language provides the determiner in a mixed DP and which provides the noun (“la store” or “the tienda,” Herring et al., 2010; see also Deuchar, 2005, 2006; Liceras et al., 2005; MacSwan, 2005; Myers-Scotton and Jake, 2015). The main hypotheses are that determiners are provided by the matrix language, in the sense of Myers-Scotton (1993), or by the language with the finer differentiation in grammatical information, for example, in grammatical gender. This question of which language provides the determiner has also been addressed experimentally from a psycholinguistic point of view (Dussias, 1997; Fairchild and van Hell, 2017). A related problem is the gender assignment of the determiner in cases when a gender-differentiating language is mixed with a language that does not differentiate gender (Liceras et al., 2005; Delgado, 2018).

The other key question with regard to CS and DPs that is directly relevant here is whether CS is more likely to occur at the determiner or at the noun, or, more generally, between phrases or within the phrase. Many studies have shown that CS between determiners and nouns is a very common phenomenon in natural speech (Timm, 1975; Pfaff, 1979; Woolford, 1983; Jake et al., 2002; Herring et al., 2010). Sankoff and Poplack (1981), who compared CS at determiners and at nouns directly, even concluded that CS is more likely to occur between determiners and nouns – that is, within phrases – than before determiners – that is, between phrases. There is also experimental evidence for the preference of CS at nouns, meaning within DPs; Dussias (1997) compared Spanish-English CS at determiners and nouns (as in *La maestra compró the/los books for the children* “The teacher bought the books for the children”). Reading times were longer for sentences with a CS at the determiner. Based on these findings, Dussias(2001, p. 98) argued that “codeswitched constituents in which functional elements do *not* participate in the codeswitching process seem to be preferred over constituents in which functional elements undergo codeswitching.”

However, not every study has found a clear preference for CS within DPs. For example, Parafita Couto and Gullberg (2019) analyzed the distribution of mixed DPs in three contact situations (Welsh-English, Spanish-English, and Papiamento-Dutch). In the two cases in which only one language figured as the matrix language (Welsh in Welsh-English CS and Papiamento in Papiamento-Dutch CS), both DPs that were switched as whole and mixed DPs were present in the corpora (in Welsh matrix sentences, there were 126 English-only DPs vs. 146 mixed DPs; in Papiamento matrix sentences, there were 66 Dutch-only DPs vs. mixed 41 DPs). Fairchild and van Hell (2017) investigated CS processing by English-speaking heritage speakers of Spanish; in a sentence-context picture-naming task, participants reacted to monolingual English (E) and Spanish (S) sentences with a final DP (EEE/SSS, respectively), sentences with CS at the determiner (ESS/SEE), and sentences with CS at nouns (EES/SSE). While the authors did not directly address the difference between all CS conditions statistically, their figures do not reveal a remarkable difference between EEE, ESS, and EES, but only longer reaction times for SSE in comparison to SEE and SSS.

To sum up, in the words of Muysken(2000, p. 5), “there is considerable variation in what is or can be inserted: in some languages this consists mostly of adverbial phrases, in others mostly single nouns, and in yet others again determiner + noun combinations.” More research is needed to investigate which factors have an impact on these preferences. Although they might be unusual for certain contact situations, the two switch points presented in Example 1 above – at a noun in a PP and at a preposition – are certainly not unusual in general terms. Their different distribution in corpus studies on CS indicates that different processes may be involved for CS at these two word classes.

### ERP Studies on Code-Switching

In this section, I discuss ERP studies that have investigated CS at the sentence level, focusing on the effect of word class/switch point. Studies on CS using ERPs differ in their experimental design and examined populations and, therefore, are not easy to compare. Not surprisingly, they also differ in the effects reported (Kutas et al., 2009; Van Hell et al., 2015, 2018). These studies almost exclusively investigated CS of single elements and most often reported on the N400 and LPC as the ERP components that reflect CS processing.

Among ERP studies on the CS of single elements, studies on the CS of nouns are prevalent. Some of these studies used visually presented stimuli in their experiments (Moreno et al., 2002; Proverbio et al., 2004), while others used auditorily presented stimuli (Liao and Chan, 2016; Ruigendijk et al., 2016; Zeller et al., 2016). The first ERP study on CS by Moreno et al.(2002; on English-Spanish CS with English-Spanish bilinguals) found a negativity for CS that was larger over the left than the right hemisphere and stretched over lateral anterior sites in contrast to a typical, non-lateralized N400. However, Proverbio et al.(2004; on English-Italian and Italian-English CS with Italian professional simultaneous interpreters), Ruigendijk et al.(2016; on German-Russian CS with Russian L1 speakers of German), Zeller et al.(2016; on Belarusian-Russian CS), and Liao and Chan(2016; on Mandarin-Taiwanese CS with Mandarin-Taiwanese bilinguals) reported a typical N400. Proverbio et al. (2004) found this N400 effect only for CS from L1 to L2, and Liao and Chan (2016) found it only for CS into the less dominant language but not vice versa.

Moreno et al. (2002); Liao and Chan (2016), and Ruigendijk et al. (2016) also observed an LPC for processing CS. This LPC varied with language proficiency: a higher proficiency led to lower LPC peaks (Moreno et al., 2002; Ruigendijk et al., 2016). An LPC was not found by Proverbio et al. (2004), which may be due to the relatively variable sentence material, the procedure (because sentences with or without CS were blocked), or the participants’ high proficiency in and experience with switching. Zeller et al. (2016) similarly did not find an LPC, which may be a consequence of either the grammatical congruency of Belarusian and Russian or the participants’ experience with frequent language mixing, which occurs in large parts of Belarusian society.

Liao and Chan (2016) manipulated not only the CS direction but also the semantic expectedness of the noun as manifested in cloze probability. For CS into the dominant language, they found an LPC only. For CS into the weaker language, they reported not only an LPC and an N400 but also a PMN (cf. Connolly and Phillips, 1994) and a long frontal negativity. The cloze probability interacted with CS only at early stages, that is, in the PMN time window (250–350 ms); there was a difference between high-cloze target switches and non-switches but not between low-cloze target switches and non-switches. The PMN for the CS of high-cloze targets corresponded to the more specific expectations of the phonological form of high-cloze words. The authors explained the long frontal negativity, which was not found in other ERP studies on intrasentential CS, as reflecting an increase of cognitive control, as had been reported in ambiguity-related studies (Lee and Federmeier, 2006; Nieuwland and Van Berkum, 2006). This increase, they argued, might be more pronounced for CS into the less dominant language because this CS direction might be less typical and, therefore, encountered less often in their participants’ daily lives.

Contrary to the studies discussed above, van der Meij et al. (2011) tested the effect of English-Spanish CS on predicative adjectives in the middle of structurally similar, visually presented sentences with Spanish learners of English. They found a typical N400 and a two-phasic LPC. The earlier part of the LPC (450–650 ms) was a broad anterior-posterior positivity, more frontal for less proficient learners, and more posterior for highly proficient learners. The later positivity (650–850 ms) was posterior, regardless of proficiency. These results are relatively similar to those of Moreno et al. (2002) and Ruigendijk et al. (2016) for CS at nouns.

Ng et al. (2014) were the first to directly compare different word classes in CS, namely nouns vs. verbs. The authors visually presented English short stories containing CS into Spanish to Spanish-English bilinguals. They found that the N400 amplitude was larger for CS at nouns than at verbs. Interestingly, an early LPC effect (i.e., following van der Meij et al., 2011) was observed but only for switched nouns, not for verbs. The authors argued that referential elements (nouns) may be harder to process and integrate than relational elements (verbs) in discourse and that the switching of nouns results in higher processing costs than the switching of verbs.

In summary, the studies on single-item CS within sentences found early negativities for CS at nouns (e.g., Liao and Chan, 2016), adjectives (van der Meij et al., 2011), and albeit less pronounced, verbs (Ng et al., 2014), which were most often interpreted as a classical N400, reflecting difficulties in processing lexical-semantic information. The direction of CS seems to be important for this component, as in some studies, it is weaker or absent for CS into the dominant language. An LPC was found for CS at nouns and adjectives but not at verbs. The LPC is sometimes identified as a member of the P300 family, reflecting the processing of a general unexpected event (cf. McCallum et al., 1984). Other researchers, such as Van Hell and Witteman (2009), see the LPC as an index of sentence-level integration and reanalysis – that is, as the language-connected P600 component (cf. Kaan et al., 2000) – or as an index of restructuring related to executive control and, hence, a more general process (Kolk and Chwilla, 2007). Following Hagoort and Brown (2000b), some researchers (van der Meij et al., 2011; Ng et al., 2014) have argued for a division of the LPC into an early subcomponent distributed both anteriorly and posteriorly and a later, clearly posterior subcomponent. Hagoort and Brown (2000b) attributed the first subcomponent to structural integration complexity and the second to (failing) parsing operations and/or reanalysis procedures. Language proficiency, CS proficiency, and structural similarity between languages seem to influence the LPC. Little attention has been paid to earlier components, and studies on processing auditory CS remain rare, but Liao and Chan (2016) found a PMN for CS of high-cloze targets, which indicates that CS may result in early extra processing costs because of violations of expectancy regarding the phonological representation of the upcoming word.

The studies discussed above did not consider the influence of the position of CS in the syntactic structure. Most used target words in the final position of the sentence (Moreno et al., 2002; Proverbio et al., 2004; Liao and Chan, 2016; Ruigendijk et al., 2016; Zeller et al., 2016). Studies that examined CS in the middle of the sentence reported comparable results (van der Meij et al., 2011; Ng et al., 2014), but because no study has compared different CS positions directly, more research is needed on the interplay of CS and syntax. Moreover, sentence structure varied across the material in Moreno et al. (2002); Proverbio et al. (2004), Ng et al. (2014), and Liao and Chan (2016), such that the switched lexical element was part of different syntactic units, including objects and PPs functioning as adjuncts. In van der Meij et al. (2011), the sentence structure remained constant, with the switched element being a predicative adjective in an embedded clause, as it was the case in Ruigendijk et al. (2016) and Zeller et al. (2016), where the switched noun was always part of a PP, denoting the direction of the action expressed by the predicate.

To the best of my knowledge, only two studies have investigated CS of larger syntactic units than single words, that is, alternational CS. Litcofsky and Van Hell (2017) examined English-Spanish bilinguals reading sentences with CS in both directions and sentences without CS. CS was at a sentence-medial noun after a determiner, most often as part of either a prepositional phrase or the direct object, and CS included not only the word in question but the remainder of the sentence. For CS into the dominant language, the authors did not find an N400 or an LPC at the switched noun; they reported an LPC for CS into the weaker language. Most importantly, they looked also at the second word in the switched block – the word after the noun (which was a function word, such as a preposition, conjunction, or determiner) – and compared this with the corresponding word in the sentence that was completely in the language of the CS. For CS into the dominant language, they reported an early (300–500 ms) anterior negativity. For CS into the weaker language, they observed a posterior positivity that continued from the first code-switched word throughout the presentation of the second code-switched word. Using the same sentence material as Litcofsky and Van Hell (2017), Fernandez et al. (2019) expanded the study of alternational CS to the auditory domain, but contrary to the earlier study, they analyzed only the first switched word. They found an N400 and an LPC for CS into the weaker language and an N400, but no LPC, for CS into the dominant language.

### The Current Study

The above-mentioned studies show that ERP studies may provide new insights into the processing of CS by distinguishing among the different aspects of language processing in CS. Hence, the aim of the current study is to examine the psycholinguistic differences in processing different types of CS using ERP, namely CS at nouns (N) in PPs and CS at prepositions (P). Comparing the effect of CS at N on the EEG with the effect of CS at P promises to shed light on the question of which effects are general for CS and which are bound to the CS of N specifically (or, in general, of open-class words). Generally, I expected CS at N and P to elicit a negativity, as well as an LPC. Based on the findings of the monolingual processing of closed-class vs. open-class words (e.g., Brown et al., 1999), as well as on CS at N vs. verbs (Ng et al., 2014), it is expected that the ERP effects of CS at N and those of CS at P differ. Because open-class words elicit an N400 pattern and CS seems to affect this pattern, it can be expected that CS at P affects the pattern elicited by closed-class items, that is, the frontal negativity found by Brown et al. (1999). However, because the prepositions used here must be classified as lexical, an N400 effect can be expected as well (cf. Chanturidze et al., 2019). If the LPC actually reflects structural integration, as argued by Hagoort and Brown (2000b), it should be modulated by the word class and, correspondingly, the syntactic position of the switched element (within an XP or at its boundary), at least in its first phase.

## Materials and Methods

### Participants

The participants were 35 L2 learners of German whose L1 was Russian. They were right-handed and had normal or corrected-to-normal vision and no known hearing deficits. Four participants were excluded because of a high number of artifacts or mistakes in the word decision task (see below) when, in at least one condition, fewer items than the mean from all participants minus 2 standard deviations could be analyzed. The final sample consisted of 25 females and six males between the ages of 19 and 35 (mean age 25.3; *SD* = 3.3). They had been living in Germany for less than one and up to 16 years (mean number of years 6.8; *SD* = 4.3), arriving in Germany between the ages of 11 and 25 (mean age 18.5; *SD* = 4.4). Some had learned German in school before arriving in Germany (four of them starting before the age of 12, that is, with 9 or 10 years). Their proficiency in German was tested with a vocabulary test (the Dialangtest)^2^. According to the Common European Framework of Reference for Languages, 21 participants were grouped as C1, seven as B2, and three as B1.

### Material

The material was based on forty quartets of German sentence contexts. Within each quartet, the verb was the same for each variant, and the nouns were closely related semantically. All followed the pattern of subject–predicate (transitive verb of motion)–direct object [see (2) below for examples and Supplementary Material for all the sentences]. The German sentences were constructed in such a way that their Russian translation equivalent would be structurally parallel to the German original. These 160 context sentences were combined with four different types of PP, which denoted the goal/direction of the denoted event. In the control condition, the whole sentence, including the PP, was in German (cf. 2a). In the second condition, the sentences ended with literal translations of the final German word, here being the lexical head of the PP, into Russian (2b). In the third condition, the complete final PP was in Russian (2c). There was a fourth condition in which the final noun was a semantically unexpected German word (e.g., *Der Bauer treibt die Kühe in diesen Schrank*, “The farmer drives the cows into this cupboard”). This fourth condition will not be analyzed in this study but has been discussed in Ruigendijk et al. (2016), which compared the processing of CS vs. semantically unexpected nouns. With this fourth condition, half of the sentences contained a CS and half were completely in German. Because the same target nouns were used in the control and in this fourth, “unexpected” condition, each German and Russian target word appeared twice in each list.

The critical words (underlined) that were triggered for EEG analysis (see below) were P in 2a and 2c and N in the final PP in 2a and 2b.

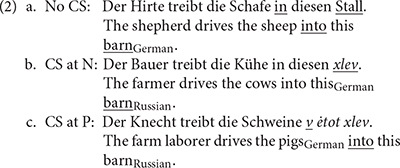

The sentence’s final Russian and German words were not cognates and were matched in number of syllables and relative word frequency^3^ using the Deutscher Wortschatz (Universität Leipzig)^4^ for German and Šarov (2001) for Russian. The PPs started either with the preposition German *in*/Russian *v* “into” or German *auf*/Russian *na* “onto,” both governing the accusative in both languages when denoting a direction. To avoid influence of gender mismatches (see section “Code-Switching in Prepositional Phrases”), German and Russian target nouns were either matched in gender (both Russian and German differentiate masculine, feminine, and neuter genders) or appeared in the plural form (because both Russian and German do not differentiate these genders in the determiner’s plural form).

Four lists were created from this material, with 160 sentences each, resulting in a total of 640 sentences. The lists differed in the combination of context sentence and type of PP to ensure that each context sentence appeared in each list, each PP appeared in each list, and across all lists all possible combinations of context sentence and target appeared only once. Stimuli were pseudo-randomized: the same condition was not to occur more than twice in a row, the language of the final word was not to be the same more than three times in a row, and the correct response to the secondary task (see below) was not to be the same more than four times in a row. The order of the context sentences in two lists was opposite to the order in the other two lists. To avoid priming effects across the four variants of each sentence quartet, there was only one variant of each sentence quartet in each of the four blocks of the experiment (see section “Procedure” below).

The 640 sentences were spoken by a female Russian-German bilingual who had shown no or hardly any recognizable accent in either of the two languages in a pretest with 12 native speakers of German and six native speakers of Russian (see Ruigendijk et al., 2016). The sentences were recorded using a Sony ECM-MS907 microphone on a MiniDisc. Afterward, they were digitized with a sample rate of 22,050 Hz as 16-bit digital sound files. The mean duration of the preposition and the following demonstrative (the time between the onsets of P and N) was 505.0 ms (*SD* = 85.4) in German and 427.2 ms (*SD* = 96.9) in Russian. This difference was significant [pairwise *t*-test: *t*(159) = 9.50, *p* < 0.001].

In a secondary task performed to ensure that the participants kept paying attention, the participants had to decide whether they had just heard a word in the sentence or not. These words were never the final word and were always in German. In 50% of the cases, the word did occur in the sentence.

### Procedure

The experiment was programmed in Eprime 2.0 (Psychology Software Tools, Pittsburgh, PA, United States). It took place in a sound-attenuating chamber. The participants were instructed that they would be listening to sentences containing German and/or Russian words. Before the experiment started, 16 sentences were presented as a practice set.

Each item started with a fixation cross that appeared on the computer screen for 1000 ms. Then, the sentence was presented through speakers (LogitechZ10). After the onset of the last word (2000 ms), a question mark appeared for 500 ms. After that, a word was presented on the screen for 1500 ms. The participants were asked to indicate whether the word had appeared in the preceding sentence by clicking the left mouse button. After a pause of 1500 ms, the next trial started automatically. The experiment consisted of four blocks of 40 sentences each. After each block, the participants could take a break and decide for themselves when they wanted to continue.

The participants performed the vocabulary test (Dialang, see above) after the experiment. Furthermore, they received a list containing all the German nouns used as target words in the experiment and were asked to check the nouns they were familiar or unfamiliar with and, if possible, to give the Russian translation. The experiment lasted around 2–2.5 h, including the preparation of electrodes and the vocabulary test.

### EEG Recording and Analysis

EEGs were recorded using 26 Ag/AgCl-electrodes attached to an elastic cap (Easycap, Munich, Germany)^5^. Electrode placement followed the International 10–20 system: F7/8, F3/4, Fz, FC5/6, FC1/2, FCz, T7/8, C3/4, Cz, CP5/6, CP1/2, P7/8, P3/4, Pz, O1/2. Signals were referenced online to the left mastoid, amplified within a bandpass of 0.01–100 Hz, and digitized at 250 Hz. The right mastoid was actively recorded, and data were re-referenced offline to the average of the left and right mastoids. Electrode impedances were kept below 3 kΩ. Eye movements were monitored by the vertical electro-oculogram (VEOG) and the horizontal electro-oculogram (HEOG).

All preprocessing was performed in EEGLAB (Delorme and Makeig, 2004). Trials were rejected automatically using the joint probability of the recorded activity (probability threshold limit of 5 standard deviations for both the single−channel and global limits) and kurtosis (local and global limits of 5 standard deviations; Delorme and Makeig, 2004). In addition, the signal was inspected by an experimenter who manually rejected trials containing artifacts. A bandpass filter of 0.05–30 Hz was also applied offline using a hamming-windowed Finite Impulse Response filter with the pop_eegfiltnew-function in EEGLAB [transition bandwidth: 0.05 Hz; filter order: 16,500; cutoff frequencies (−6 dB): 0.025 and 30.025 Hz]. For illustrative purposes, ERPs were computed by averaging the EEG per condition for each subject at each electrode site for a time window from 200 ms prior to the onset of the critical words to 1200 ms after the onset, which signifies the P in 2a and 2c and the N in the final PP in 2a and 2b. A baseline correction was carried out using the 200 ms prior to the onset of the critical word.

The mean number of trials that entered the averaging process and statistical analyses were as follows: 35.7, *SD* = 2.7 (no CS, time-locked to N, 2a), 36.3, *SD* = 2.0 (no CS, time-locked to P, 2a), 36.1, *SD* = 2.7 (CS at N, 2b), and 35.5, *SD* = 2.8 (CS at P, 2c). All statistical analyses were performed in R (R Core Team, 2020) using the lme4-package (Bates et al., 2015b) and lmerTest-package (Kuznetsova et al., 2017). Although the participants varied in German proficiency as their L2, which arguably has an influence on the processing of CS, language proficiency was not included as a factor in the model for two reasons. First, this study is primarily interested in the general impact of word class and switch point on the processing of CS. Second, this additional factor would further complicate the statistical models, both conceptually and computationally. To keep the statistical models simple, I also decided to focus on the anteriority-posteriority dimension and not include laterality as a factor. The electrodes were therefore averaged in the following two regions of interest: anterior (F3/4, Fz, FC1/2, FC5/6) and posterior (CP1/2, CP5/6, P3/4, Pz).

**FIGURE 1:**
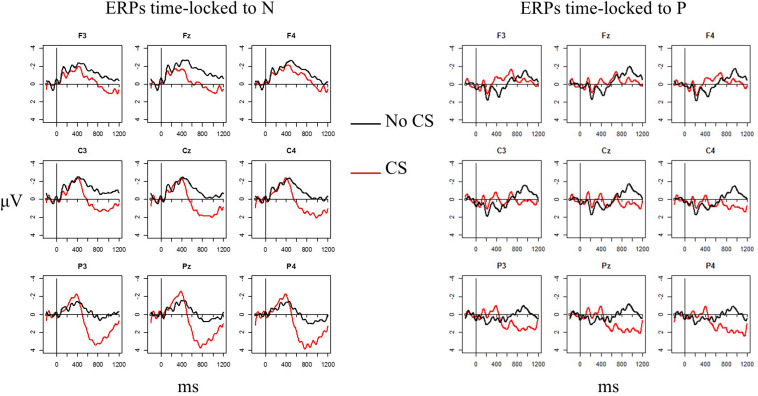
Grand average waveforms time-locked to N (left) and P (right) for selected electrodes. Waveforms for CS are in red, and waveforms for no CS are in black.

Figure 1 suggests that CS at N elicited one negative component around 400 ms (in line with previous studies, but see below), whereas CS at P elicited not only a negative component around 400 ms, but also an even earlier negativity between 100 and 200 ms (in line with CS of high-cloze targets in Liao and Chan, 2016). To examine these different effects, for each trial the mean amplitudes were calculated in two early time windows, time-locked to P or N, respectively, a first one from 100 to 200 ms and a second one from 200 to 500 ms. Following van der Meij et al. (2011), who divided the late time window targeting the LPC in two, the mean amplitudes were also calculated in time windows of 500–800 ms and 800–1000 ms. A linear mixed-effects model was calculated for each time window, with Subject (*n* = 31) and Item (*n* = 160) as random factors and Anteriority (posterior vs. anterior), Point (N vs. P), CS (CS vs. no CS), and their interactions as fixed effects. Anteriority, Point, and CS were deviation coded. Following Barr (2013) and Barr et al. (2013), I began with models that included the full random effects structure, including random slopes for the highest-order interaction Anteriority × Point × CS. These models did not converge. A principal component analysis using the rePCA-function in the lme4-package also showed that these models were over-parameterized. Random slopes for Anteriority were excluded from the models after inspection of the variance of the random slopes, following Barr et al. (2013) and Bates et al. (2015a). When the number of parameters was still not supported by the data and the models did not converge, the random effects structure was further reduced by excluding random slopes, based again on the results of principal component analyses. The final model for the first time window (100–200 ms) included random slopes for CS and Point per Subject, as well as for CS, Point, and the CS × Point interaction per Item. For the second and third time window (200–500 ms and 500–800 ms), the final model included random slopes for CS, Point, and the CS × Point interaction per Subject, as well as for CS and Point per Item. For the last time window (800–1000 ms), the final model included random slopes for CS, Point, and the CS × Point interaction both per Subject and per Item. Finally, after recoding the data and excluding non-significant interactions, relevant contrasts (CS vs. no CS) were investigated with the help of simple effects analyses. All final models can be found in Supplementary Material.

## Results

### Familiarity With the Target Words and Word Monitoring

On average, the participants were unfamiliar with or mistranslated 1.6 German target nouns (σ = 1.7, range: 0–7). In the word monitoring task, the participants scored on average 117.8 out of 120 (σ = 1.8, range: 114–120); for the no-CS control condition (2a): 39.6 (σ = 0.7, 38–40); for CS at N (2b): 39.5 (σ = 0.7, 37–40), and for CS at P (2c): 38.7 (σ = 1.5, 35–40). This indicates that the participants were generally familiar with the German target nouns and listened to the sentences carefully.

### Effects of Word Class on Processing CS: ERP Analysis

**FIGURE 2:**
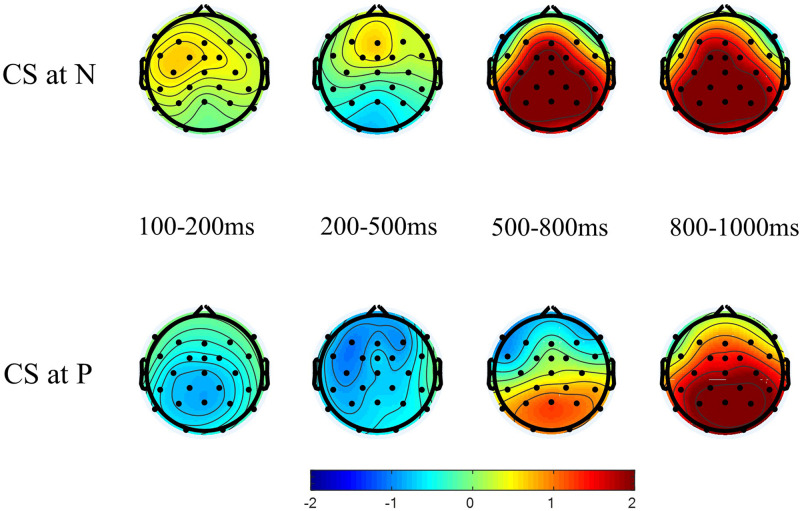
Scalp topography of the CS effect (CS condition minus control condition) in the four tested time windows for the two switch points, N (upper panel) and P (lower panel).

Figure 1 shows the grand average waveforms for no CS at N vs. CS at N and for no CS at P vs. CS at P for a selection of electrodes. Figure 2 shows the scalp topography of CS processing for the two switch points (CS condition minus control condition) for the four tested time windows.

Visual inspection suggested a broadly distributed negative component for CS at P and a posterior negative component for CS at N in the two early time windows. In the third time window, visual inspection suggested a broadly distributed positivity that is strongest at posterior sites for CS at N and a weaker posterior positivity, together with an anterior negativity, for CS at P. In the fourth time window, the figures reveal a broadly distributed positivity that is strongest at the posterior sites for CS at both N and P.

Below, I report the analyses per time window. Only the main effects of CS and Point, as well as the interactions between CS and Point, are reported. The complete outcome of the models can be found in Supplementary Material.

#### Early Time Window 1 (100–200 ms)

In this first time window, there was a main effect for Point, with less negative ERPs for P compared to N [*b* = 1.33, *SE* = 0.32, *t*(48.14) = 4.20, *p* < 0.001]. Point interacted with Anteriority [*b* = 0.65, *SE* = 0.27, *t*(8171) = 2.45, *p* = 0.014]; for N, but not for P, the ERP was more negative anteriorly than posteriorly. There was no main effect for CS [*b* = −0.11, *SE* = 0.24, *t*(47.22) = −0.44, *p* = 0.661], but an interaction occurred between CS and Point, with CS at P eliciting more negative responses than CS at N [*b* = −0.97, *SE* = 0.40, *t*(157.50) = −2.44, *p* = 0.016]. Anteriority did not interact with CS [*b* = 0.30, *SE* = 0.27, *t*(8171) = 1.13, *p* = 0.258], and there was no three-way interaction between Anteriority, CS, and Point [*b* = 0.01, *SE* = 0.53, *t*(8171) = 0.02, *p* = 0.987]. This confirms that the CS effect was different for N and P, but this difference was comparable anteriorly and posteriorly. Simple effects analyses revealed that there was an effect of CS at P with more negative ERPs [*b* = −0.59, *SE* = 0.30, *t*(88.45) = −2.00, *t* = 0.049]. For N, there was no effect of CS [*b* = 0.38, *SE* = 0.32, *t*(106.11) = 1.18, *p* = 0.243].

#### Early Time Window 2 (200–500 ms)

In the second early time window, there was a main effect for Point, caused by more positive responses for P [*b* = 2.17, *SE* = 0.34, *t*(46.23) = 6.31, *p* < 0.001], and an interaction between Anteriority and Point [*b* = 0.57, *SE* = 0.27, *t*(8308.82) = 2.09, *p* = 0.037]. The ERPs in reaction to N were more negative at anterior electrodes compared to posterior electrodes, whereas there was no such difference for P. There was no main effect for CS [*b* = −0.39, *SE* = 0.25, *t*(43.47) = −1.57, *p* = 0.123], no interaction between CS and Anteriority [*b* = 0.24, *SE* = 0.27, *t*(8308.82) = 0.90, *p* = 0.371], and no interaction between CS and Point [*b* = −0.84, *SE* = 0.49, *t*(29.71) = −1.70, *p* = 0.100]. The three-way interaction between Anteriority, CS, and Point reached marginal significance [*b* = −0.91, *SE* = 0.54, *t*(8308.82) = −1.68, *p* = 0.093]. Following up on this marginally significant interaction, simple effects analyses revealed that CS at P resulted in a negativity at anterior electrodes [*b* = −0.92, *SE* = 0.38, *t*(69.31) = −2.43, *p* = 0.018]. At anterior electrodes, the difference between the CS effect for N and P was significant as well, confirming that the CS effect at frontal sites was less negative for N [*b* = 1.29, *SE* = 0.56, *t*(50.44) = 2.30, *p* = 0.026]. At posterior electrodes, the CS effect at P was marginally significant [*b* = −0.70, *SE* = 0.38, *t*(69.31) = −1.87, *p* = 0.066] and did not differ from the CS effect at N [*b* = 0.38, *SE* = 0.56, *t*(50.44) = 0.68, *p* = 0.501]. The simple effects models did not converge with N and no CS as the reference values, so CS and N were taken as the reference values. Non-switched N did not elicit more positive ERPs than switched N, neither at posterior electrodes [*b* = 0.32, *SE* = 0.42, *t*(56.31) = 0.77, *p* = 0.447] nor at anterior electrodes [*b* = −0.38, *SE* = 0.42, *t*(56.31) = −0.89, *p* = 0.376].

#### Late Time Window 1 (500–800 ms)

In the first LPC time window, there was no main effect for Point [*b* = 0.40, *SE* = 0.35, *t*(42.62) = 1.17, *p* = 0.249], but there was an interaction between Point and Anteriority [*b* = 0.93, *SE* = 0.31, *t*(8306.93) = 2.99, *p* = 0.003]. For both N and P, the ERPs were more positive at posterior electrodes, but this difference was more pronounced for N. There was a main effect for CS [*b* = 1.00, *SE* = 0.36, *t*(42.95) = 2.81, *p* = 0.007], caused by ERPs that were on average more positive for switches than non-switches. CS interacted with Anteriority to the extent that the CS effect was less positive at anterior electrodes [*b* = −1.20, *SE* = 0.31, *t*(8306.93) = −3.89, *p* < 0.001]. There was an interaction between CS and Point: CS at P resulted in less positive ERPs compared to CS at N [*b* = −1.56, *SE* = 0.56, *t*(29.58) = −2.76, *p* = 0.010]. There was no three-way interaction between Anteriority, CS, and Point [*b* = −0.12, *SE* = 0.62, *t*(8306.93) = −0.19, *p* = 0.850].

Simple effects analyses revealed no difference between switched and non-switched P at anterior electrodes [*b* = −0.38, *SE* = 0.41, *t*(55.83) = −0.92, *p* = 0.362], but there was a difference at posterior electrodes [*b* = 0.83, *SE* = 0.41, *t*(55.83) = 2.01, *p* = 0.049]. Compared to non-switched N, switched N resulted in more positive ERPs at both anterior [*b* = 1.18, *SE* = 0.54, *t*(42.27) = 2.18, *p* = 0.035] and posterior electrodes [*b* = 2.38, *SE* = 0.54, *t*(42.27) = 4.40, *p* < 0.001]. The CS effect was more positive at posterior electrodes than at anterior electrodes for both P and N [*b* = 1.20, *SE* = 0.31, *t*(8307.93) = 3.89, *p* < 0.001].

#### Late Time Window 2 (800–1000 ms)

In the second LPC time window, there was a main effect for Point, with more negative ERPs for prepositions on average [*b* = −0.72, *SE* = 0.28, *t*(56.67) = −2.60, *p* = 0.012]. Point interacted with Anteriority, as the difference between N and P was less pronounced at anterior sites [*b* = 0.72, *SE* = 0.33, *t*(8143.04) = 2.19, *p* = 0.029]. Again, the ERPs for both N and P were more positive at posterior electrodes, but this difference was bigger for N. There was a main effect for CS: in general, CS resulted in more positive responses than non-switches [*b* = 1.66, *SE* = 0.36, *t*(44.01) = 4.60, *p* < 0.001]. There was also an interaction between CS and Anteriority, confirming that the CS effect was less strong at anterior electrodes than at posterior electrodes [*b* = −1.07, *SE* = 0.33, *t*(8143.04) = −3.25, *p* = 0.001]. There was no interaction between CS and Point [*b* = −0.07, *SE* = 0.48, *t*(60.44) = −0.15, *p* = 0.878], nor was there an interaction between CS, Point, and Anteriority [*b* = −0.35, *SE* = 0.66, *t*(8143.04) = −0.53, *p* = 0.600]. This indicates that the CS effect in this time window was comparable for P and N.

Simple effects analyses revealed CS effects at anterior [*b* = 1.13, *SE* = 0.40, *t*(64.92) = 2.86, *p* = 0.006] and posterior electrodes [*b* = 2.20, *SE* = 0.40, *t*(64.92) = 5.56, *p* < 0.001], with the positivity being more pronounced at posterior sites [*b* = 1.07, *SE* = 0.33, *t*(8329.46) = 3.22, *p* = 0.001].

To sum up, CS at N had no significant effect in the two early time windows, but resulted in a broad, posteriorly centered positivity in the later time windows. CS at P, in contrast, resulted in a broad negativity in the two early time windows, followed by an anterior negativity with a posterior positivity in the third, and a broad, posteriorly centered positivity in the last window.

## Discussion

The current study aimed to ascertain whether the processing of CS differs depending on word class and, specifically, whether the switch takes place at a noun or at a preposition. For CS at nouns, the overall results were similar to those of other ERP studies that examined CS at content words. More precisely, CS at nouns in the current study showed similarities both to studies that, like the current one, examined CS at nouns in a sentence-final position (Moreno et al., 2002; Liao and Chan, 2016) and to the results of van der Meij et al. (2011), who examined CS at adjectives in the middle of the sentence. CS at prepositions elicited ERPs that in some aspects differed from those elicited by CS at content words, but they showed some similarities to the results reported by Liao and Chan (2016), who also studied CS at nouns but manipulated the semantic expectedness of the switched word. In particular, the results for the early time windows (100–200 ms, 200–500 ms) and the first late time window (500–800 ms) show that the processing of CS at a noun compared with CS at a preposition is different; that is, the effect of CS is modulated by the word class of the switched element. There was no such modulation in the second late time window (800–1000 ms), indicating that there are late processes connected with CS that are independent of word class.

I will first discuss the second early time window (200–500 ms), associated with the N400. In this time window, the processing of CS at a preposition resulted in a significant anterior negativity and a marginally significant posterior negativity. This was observed following up an only marginally significant interaction between Anteriority, CS, and Point. Nevertheless, I would argue that the posterior negativity for CS at prepositions could be classified as a typical N400 effect (as in Kutas and Hillyard, 1980). N400 effects similar to those elicited by grammatically correct but unexpected words have been found frequently for CS of nouns and other lexical words. Because violations of lexical prepositions also elicit N400 effects (Chanturidze et al., 2019), the N400 effect for CS of prepositions found in the present study can be seen as a parallel to the N400 effect frequently found for CS at nouns.

However, this effect was only marginally significant, and there was no corresponding significant N400 effect for CS at nouns, although the waveforms and topographies in Figures 1, 2 resemble the typical N400 pattern. The N400 effect is found frequently in ERP studies on CS but by no means in every study, especially not for CS into the more dominant language, as was the case in the current study. Proverbio et al. (2004) found an N400 effect only for CS from L1 into L2, and Liao and Chan (2016) only for CS into the less dominant language but not vice versa. I would argue that the presence or absence of an N400 effect relates to the extent to which listeners are able to use the linguistic context in their less dominant language to predict upcoming words. Note that in the current study proficiency was not included as a factor in the statistical models. Using the same data, Ruigendijk et al. (2016) differentiated between highly and moderately proficient L2 learners when they compared processing CS at nouns with processing nouns that were semantically unexpected. They found an N400 effect for CS at nouns only with highly proficient L2 learners. The lack of an N400 with the moderately proficient learners was caused by the relatively negative ERPs for nouns in the German control sentences. Ruigendijk et al. (2016) argued that moderately proficient L2 learners were less able to use the context to pre-activate an upcoming word. Their findings align with those of Hahne (2001), who observed a larger N400 in regular sentences for L2 learners compared to native speakers, and of Martin et al. (2013), who argued that L2 speakers are less able to predict upcoming words in a constrained sentence than L1 speakers. Therefore, it follows that in the present study the N400 effect is more pronounced (albeit still weak) for switched prepositions because prepositions as closed-class items are arguably more predictable than nouns even for moderate L2 learners.

The broad negativity in the first early time window (100–200 ms) and the anterior negativity in the second early time windows for CS at prepositions should be characterized differently from the N400-like posterior negativity. Arguably, it should also be explained differently for the first and second early time windows (in line with Liao and Chan, 2016). I will start with discussion of the first early time window. Studies on the auditory processing of semantic violations have regularly noticed earlier negativities than studies on the visual processing of such violations. Some have interpreted this early negativity as an early onset of the N400 (Van Petten et al., 1999; Van Den Brink and Hagoort, 2004; Diaz and Swaab, 2007), while others have argued for a different functional explanation. Following Connolly and Phillips (1994), Hagoort and Brown(2000a, p. 1528) argued that the early negativity is a PMN, that is, an index of the early detection of “a phonological mismatch between the actual word and the expected lexical candidate.” This conclusion is supported by the fact that the early negativity was not found in the study by Friederici et al. (1993) in which the target word was unexpected on semantic grounds but had the same onset as the expected word. Therefore, it follows that CS at prepositions elicits this negativity more than CS at nouns because, in general, prepositions (as closed-class elements), including their phonological form, can be predicted with a higher probability than a specific noun. Under these circumstances, the onset of a switched preposition would be more surprising and lead to higher detection costs than the onset of a switched noun.

As for the second early time window, the anterior negativity for CS at prepositions is in line with the observation that prepositions and closed-class items generally elicit an anterior negativity compared with open-class items, probably because they serve as a syntactic signal that a new head/meaningful word is coming up (Van Petten and Kutas, 1991; Brown et al., 1999). The anterior negativity in this time window may be connected to the increased effort in processing this signal because of CS. An alternative interpretation is that the anterior negativity reflects an increase in cognitive control; Liao and Chan (2016) found a frontal negativity only for CS into the participants’ less dominant language and argued that higher costs in cognitive control might be caused by the lower typicality and frequency of this CS direction. In the present study, CS occurred only into the participants’ dominant language, but one might speculate whether a general lower probability of CS at prepositions compared with CS at nouns (see section “Code-Switching in Prepositional Phrases”) might have caused an increase in cognitive control for CS at prepositions. More research is needed to account for this effect.

As for the late time windows, there was a typical LPC for CS at nouns in both time windows. In the first of these two late time windows (500–800 ms), CS at prepositions differed from CS at nouns, resulting in a less strong posterior LPC and even in a frontal negativity. This supports the interpretation that for this time window, the LPC is not an index of “surprise,” that is, of the detection of a surprising form, as Moreno et al. (2002) have argued (in fact, the first early time window seems to be a more appropriate candidate for this, as discussed above). The “surprise” should be comparable for both word classes or higher for CS at prepositions (see section “Code-Switching in Prepositional Phrases”). Following Hagoort and Brown (2000b), who attributed the first subcomponent of the LPC to structural integration complexity, structural aspects should be responsible for the difference between CS at the two word classes in this time window. This ratio suggests that CS at phrase boundaries (in this case, at the preposition of a PP) is easier to process than CS within an XP (in this case, at a noun within a PP). However, this would be in contradiction with the fact that nouns within DPs are switched easily (see section “Code-Switching in Prepositional Phrases”), as well as with the results of Dussias (1997), who reported higher processing costs for CS at phrase boundaries than for CS within phrases. A factor that might interact here is the syntactic and functional status of the DP in the sentence. CS between direct objects and locative phrases, as in the current study, may be easier to process than CS between, for instance, the predicate and the direct object. Note also that the component discussed here is only one of several components engaged in processing CS, so the fact that it is less pronounced for CS at prepositions does not necessarily indicate that CS at a preposition is easier to process than CS at nouns in general.

In the last time window, no differences were found between the LPC for CS at nouns and prepositions. This indicates that this part of the LPC is independent from or at least less sensitive to the structural aspects of CS, but it may be connected with general reanalysis procedures (cf. Hagoort and Brown, 2000b) elicited by the presence of two languages in one sentence.

## Conclusion

CS includes a wide and heterogeneous set of phenomena. The current study used ERPs to examine the differences in the processing of different manifestations of CS when switching from the L2 into the L1. The results show that psycholinguistic processes in CS are more heterogeneous and complex than ERP studies have suggested so far. Indeed, ERP studies still have much to contribute to our understanding of these phenomena.

Although it is clear that CS at nouns and prepositions is processed differently, it is hard to say whether CS at nouns or prepositions is easier to process. In fact, this question must be posed more specifically by taking into account that different subprocesses are at work when processing CS.

The psycholinguistic differences in processing CS of nouns and CS of prepositions, as revealed by ERPs, can be related to the following:

• General differences in processing these word classes, that is, word-class–specific components, such as the anterior negativity for prepositions;
• Differences between open-class elements and closed-class elements in the predictability of lexical items, including their phonological form;
• Differences in the structural position that nouns and prepositions have in a sentence.

It is also important to note that some processes seem to be similar for CS at nouns and CS at prepositions. This makes sense as both are CS and thus manifestations of the same general phenomenon, so they can be expected to share some common features. This is the case for the second phase of the LPC, which can be attributed to reanalysis procedures, which are, in this case, elicited by the detection of a discrepancy between the language of the processed element and the previous context.

## Data Availability Statement

The datasets generated for this study are available on request to the corresponding author.

## Ethics Statement

The studies involving human participants were reviewed and approved by the Kommission für Forschungsfolgenabschätzung und Ethik, University of Oldenburg. The patients/participants provided their written informed consent to participate in this study.

## Author Contributions

JZ contributed to the conception and design of the study, performed the statistical analysis, and wrote all sections of the manuscript.

## Conflict of Interest

The author declares that the research was conducted in the absence of any commercial or financial relationships that could be construed as a potential conflict of interest.

## Footnotes

1 A four-way distinction would include prepositions indicating a structural position in the clause (e.g., *by* as the marker for the demoted agent in passive clauses), lexically governed prepositions (like *on* in *to rely on someone*), idiosyncratically or metaphorically used prepositions (like in *in the mood*), and fully lexical prepositions that stand in paradigmatic opposition to other prepositions and contribute to the semantics of the clause (like in *on/under/in front of the table*; Hentschel, 2003).

2 Current location: https://dialangweb.lancaster.ac.uk/ (retrieved September 10, 2019).

3 Relative to the most frequent word in the language (i.e., the masculine definite determiner *der* “the” in German; and *i* “and” in Russian).

4 Current location: https://wortschatz.uni-leipzig.de/en (retrieved September 10, 2019).

5 www.easycap.de

## References

[B1] Backus A. (1996). *Two in One. Bilingual Speech of Turkish Immigrants in the Netherlands.* Tilburg: Tilburg University Press.

[B2] Barr D. J. (2013). Random effects structure for testing interactions in linear mixed-effects models. *Front. Psychol.* 4:328. 10.3389/fpsyg.2013.00328 23761778 PMC3672519

[B3] Barr D. J. Levy R. Scheepers C. Tily H. J. (2013). Random effects structure for confirmatory hypothesis testing: keep it maximal. *J. Mem. Lang.* 68 255–278. 10.1016/j.jml.2012.11.001 24403724 PMC3881361

[B4] Bates D. Kliegl R. Vasishth S. Baayen H. (2015a). *Parsimonious Mixed Models.* Available online at: https://arxiv.org/abs/1506.04967 (accessed May 26, 2018).

[B5] Bates D. Maechler M. Bolker B. Walker S. (2015b). Fitting Linear Mixed-Effects Models Using lme4. *J. Stat. Softw.* 67 1–48.

[B6] Bentahila A. Davies E. E. (1983). The syntax of Arabic-French code-switching. *Lingua* 59 301–330. 10.1016/0024-3841(83)90007-4

[B7] Berk-Seligson S. (1986). Linguistic constraints on intrasentential code-switching: a study of Spanish/Hebrew bilingualism. *Lang. Soc.* 15 313–348. 10.1017/s0047404500011799

[B8] Brown C. M. Hagoort P. Ter Keurs M. (1999). Electrophysiological signatures of visual lexical processing: open-and closed-class words. *J. Cogn. Neurosci.* 11 261–281. 10.1162/089892999563382 10402255

[B9] Chanturidze M. Carroll R. Ruigendijk E. (2019). Prepositions as a hybrid between lexical and functional category: evidence from an ERP study on German sentence processing. *J. Neurolinguist.* 52:100857 10.1016/j.jneuroling.2019.100857

[B10] Clyne M. (1987). Constraints on code switching: how universal are they? *Linguistics* 25 739–764.

[B11] Connolly J. F. Phillips N. A. (1994). Event-related potential components reflect phonological and semantic processing of the terminal word of spoken sentences. *J. Cogn. Neurosci.* 6 256–266. 10.1162/jocn.1994.6.3.256 23964975

[B12] Corver N. van Riemsdijk H. (eds) (2001). *Semi-Lexical Categories: The Function of Content Words and the Content of Function Words.* Berlin: Walter de Gruyter.

[B13] Delgado R. (2018). “The familiar and the strange. Gender assignment in Spanish/English mixed DPs,” in *Code-Switching – Experimental Answers to Theoretical Questions: In Honor of Kay González-Vilbazo*, ed. López L. (Amsterdam: John Benjamins), 39–62. 10.1075/ihll.19.03del

[B14] Delorme A. Makeig S. (2004). ‘EEGLAB: an open source toolbox for analysis of single-trial EEG dynamics‘. *J. Neurosci. Methods* 134 9–21. 10.1016/j.jneumeth.2003.10.009 15102499

[B15] Deuchar M. (2005). Congruence and code-switching in Welsh. *Bilingualism* 8 1–15.

[B16] Deuchar M. (2006). Welsh–English code-switching and the Matrix Language Frame model. *Lingua* 116 1986–2011. 10.1016/j.lingua.2004.10.001

[B17] Di Sciullo A. Muysken P. Singh R. (1986). Government and code-mixing. *J. Linguist.* 22 1–24. 10.1017/s0022226700010537

[B18] Diaz M. T. Swaab T. Y. (2007). Electrophysiological differentiation of phonological and semantic integration in word and sentence contexts. *Brain Res.* 1146 85–100. 10.1016/j.brainres.2006.07.034 16952338 PMC1853329

[B19] Dussias P. E. (1997). Sentence matching and the functional head constraint in Spanish/English codeswitching. *Spanish Appl. Linguist.* 1 114–150.

[B20] Dussias P. E. (2001). Psycholinguistic complexity in codeswitching. *Int. J. Bilingualism* 5 87–100. 10.1177/13670069010050010501

[B21] Fairchild S. van Hell J. (2017). Determiner-noun code-switching in Spanish heritage speakers. *Bilingualism* 20 150–161. 10.1017/s1366728915000619

[B22] Fernandez C. B. Litcofsky K. A. Van Hell A. (2019). Neural correlates of intra-sentential code-switching in the auditory modality. *J. Neurolinguist.* 51 17–41. 10.1016/j.jneuroling.2018.10.004

[B23] Friederici A. D. Pfeifer E. Hahne A. (1993). Event-related brain potentials during natural speech processing: effects of semantic morphological syntactic violations. *Cogn. Brain Res.* 1 183–192. 10.1016/0926-6410(93)90026-2 8257874

[B24] Gardner-Chloros P. (2009). *Code-Switching.* Cambridge: Cambridge University Press.

[B25] Hagoort P. Brown C. M. (2000a). ERP effects of listening to speech: semantic ERP effects. *Neuropsychologia* 38 1518–1530. 10.1016/s0028-3932(00)00052-x 10906377

[B26] Hagoort P. Brown C. M. (2000b). ERP effects of listening to speech compared to reading: the P600/SPS to syntactic violations in spoken sentences and rapid serial visual presentation. *Neuropsychologia* 38 1531–1549. 10.1016/s0028-3932(00)00053-1 10906378

[B27] Hahne A. (2001). What’s different in second-language processing? Evidence from event-related brain potentials. *J. Psycholinguist. Res.* 30 251–266. 11523274 10.1023/a:1010490917575

[B28] Hentschel G. (2003). “Zur Klassifikation von Präpositionen im Vergleich zur Klassifikation von Kasus,” in *Präpositionen im Polnischen. Beiträge zu einer gleichnamigen Tagung, Oldenburg, 8. bis 11*, eds Hentschel G. Menzel T. (Oldenburg: BIS), 161–191.

[B29] Herring J. R. Deuchar M. Parafita Couto M. C. Moro Quintanilla M. M. (2010). ‘I saw the madre’: evaluating predictions about codeswitched determiner noun sequences using Spanish–English and Welsh–English data. *J. Bilingual Educ. Bilingualism* 13 553–573. 10.1080/13670050.2010.488286

[B30] Hillyard S. A. (1973). The CNV and human behavior: a review. *Electroencephalogr. Clin. Neurophysiol.* 33 161–171.

[B31] Jake J. L. Myers-Scotton C. Gross S. (2002). Making a minimalist approach to codeswitching work: adding the Matrix Language. *Bilingualism Lang. Cogn.* 5 69–91.

[B32] Joshi A. K. (1984). “Processing of sentences with intra-sentential code-switching,” in *Natural Language Processing: Psychological, Computational and Theoretical Perspectives*, eds Douty D. Kartunen L. Zwicky A. (Cambridge: Cambridge University Press), 145–150.

[B33] Kaan E. Harris A. Gibson E. Holcomb P. J. (2000). The P600 as an index of syntactic integration difficulty. *Lang. Cogn. Process.* 15 159–201. 10.1080/016909600386084

[B34] Kolk H. Chwilla D. (2007). Late positivities in unusual situations. *Brain Lang.* 100 257–261. 10.1016/j.bandl.2006.07.006 16919324

[B35] Kutas M. Federmeier K. D. (2000). Electrophysiology reveals semantic memory use in language comprehension. *Trends Cogn. Sci.* 4 463–470. 10.1016/s1364-6613(00)01560-6 11115760

[B36] Kutas M. Hillyard S. A. (1980). Reading senseless sentences: brain potentials reflect semantic incongruity. *Sci. New Ser.* 207 203–205. 10.1126/science.7350657 7350657

[B37] Kutas M. Moreno E. M. Wicha N. (2009). “Code-switching and the brain,” in *The Cambridge Handbook of Linguistic Code-Switching*, eds Bullock B. E. Toribio A. J. (Cambridge: Cambridge University Press), 289–306. 10.1017/cbo9780511576331.018

[B38] Kuznetsova A. Brockhoff P. B. Christensen R. H. B. (2017). lmerTest package: tests in linear mixed effects models. *J. Stat. Softw.* 82 1–26.

[B39] Lee C.-L. Federmeier K. D. (2006). To mind the mind: an event-related potential study of word class and semantic ambiguity. *Brain Res.* 1081 191–202. 10.1016/j.brainres.2006.01.058 16516169 PMC2728580

[B40] Liao C. H. Chan S. H. (2016). Direction matters: event-related brain potentials reflect extra processing costs in switching from the dominant to the less dominant language. *J. Neurolinguist.* 40 79–97. 10.1016/j.jneuroling.2016.06.004

[B41] Liceras J. M. Spradlin K. T. Fuertes R. F. (2005). Bilingual early functional-lexical mixing and the activation of formal features. *Int. J. Bilingualism* 9 227–252. 10.1177/13670069050090020601

[B42] Lipski J. M. (1977). “Code-switching and the problem of bilingual competence,” in *The Fourth LACUS forum*, ed. Paradis M. (Columbia, SC: Hornbeam Press), 250–264.

[B43] Litcofsky K. A. Van Hell J. G. (2017). Switching direction affects switching costs: behavioral, ERP and time-frequency analyses of intra-sentential codeswitching. *Neuropsychologia* 97 112–139. 10.1016/j.neuropsychologia.2017.02.002 28167120

[B44] MacSwan J. (2005). Codeswitching and generative grammar: a critique of the MLF model and some remarks on ‘modified minimalism’. *Bilingualism* 8 1–22. 10.1017/s1366728904002068

[B45] Martin C. D. Thierry G. Kuipers J.-R. Boutonnet B. Foucart A. Costa A. (2013). Bilinguals reading in their second language do not predict upcoming words as native readers do. *J. Mem. Lang.* 69 574–588. 10.1016/j.jml.2013.08.001

[B46] Matras Y. (2009). *Language Contact.* Cambridge: Cambridge University Press.

[B47] McCallum W. C. Farmer S. F. Pocock P. V. (1984). The effects of physical and semantic incongruities of auditory event-related potentials. *Electroencephalogr. Clin. Neurophysiol.* 59 477–488. 10.1016/0168-5597(84)90006-6 6209114

[B48] Milroy L. Muysken P. (1995). “Introduction: code-switching and bilingualism research,” in *One Speaker, Two Languages: Cross-Disciplinary Perspectives on Code-Switching*, eds Milroy L. Muysken P. (Cambridge: Cambridge University Press), 1–14. 10.1017/cbo9780511620867.001

[B49] Moreno E. M. Federmeier K. D. Kutas M. (2002). Switching languages, switching palabras (words): An electrophysiological study of code switching. *Brain Lang.* 80 188–207. 10.1006/brln.2001.2588 11827443

[B50] Muysken P. (2000). *Bilingual Speech: A Typology of Code Mixing.* Cambridge: Cambridge University Press.

[B51] Myers-Scotton C. (1993). *Duelling Languages: Grammatical Structure in Codeswitching.* Oxford: Oxford University Press.

[B52] Myers-Scotton C. (2006). Natural codeswitching knocks on the laboratory door. *Bilingualism* 9 203–212. 10.1017/s1366728906002549

[B53] Myers-Scotton C. M. Jake J. L. (2015). “Cross-language asymmetries in code-switching patterns: implications for bilingual language production,” in *The Cambridge Handbook of Bilingual Processing*, ed. Schwieter J. W. (Cambridge: Cambridge University Press), 416–458. 10.1017/cbo9781107447257.019

[B54] Ng S. Gonzalez C. Wicha N. (2014). The fox and the cabra: an ERP analysis of reading code switched nouns and verbs in bilingual short stories. *Brain Res.* 1557 127–140. 10.1016/j.brainres.2014.02.009 24530431 PMC3982600

[B55] Nieuwland M. S. Van Berkum J. J. (2006). Individual differences and contextual bias in pronoun resolution: evidence from ERPs. *Brain Res.* 1118 155–167. 10.1016/j.brainres.2006.08.022 16956594

[B56] Parafita Couto M. C. Gullberg M. (2019). Code-switching within the noun phrase: Evidence from three corpora. *Int. J. Bilingualism* 23 695–714. 10.1177/1367006917729543

[B57] Pfaff C. W. (1979). Constraints on language mixing: intrasentential code-switching and borrowing in Spanish/English. *Language* 55 291–318.

[B58] Proverbio A. M. Leoni G. Zani A. (2004). Language switching mechanisms in simultaneous interpreters: an ERP study. *Neuropsychologia* 42 1636–1656. 10.1016/j.neuropsychologia.2004.04.013 15327931

[B59] R Core Team (2020). *R: A Language and Environment for Statistical Computing.* Vienna: R Foundation for Statistical Computing.

[B60] Ruigendijk E. Hentschel G. Zeller J. P. (2016). How L2-learners’ brains react to code switches. An ERP study with Russian learners of German. *Sec. Lang. Res.* 32 197–223. 10.1177/0267658315614614

[B61] Sankoff D. Poplack S. (1981). A formal grammar for code-switching 1. *Res. Lang. Soc. Interact.* 14 3–45. 10.1080/08351818109370523

[B62] Šarov S. A. (2001). *Èastotnyj Slovar’.* Available online at: http://www.artint.ru/projects/frqlist.php/ (accessed September 10, 2018).

[B63] Timm L. A. (1975). Spanish–English code-switching: el porque y how-not-to. *Romance Philol.* 28 473–482.

[B64] Van Den Brink D. Hagoort P. (2004). The influence of semantic and syntactic context constraints on lexical selection and integration in spoken-word comprehension as revealed by ERPs. *J. Cogn. Neurosci.* 16 1068–1084. 10.1162/0898929041502670 15298793

[B65] van der Meij M. Cuetos F. Carreiras M. Barber H. A. (2011). Electrophysiological correlates of language switching in second language learners. *Psychophysiology* 48 44–54. 10.1111/j.1469-8986.2010.01039.x 21143487

[B66] Van Hell J. Litcofsky K. Ting C. (2015). “Intra-sentential code-switching,” in *The Cambridge Handbook of Bilingual Processing*, ed. Schwieter J. (Cambridge: Cambridge University Press), 459–482.

[B67] Van Hell J. G. Fernandez C. B. Kootstra G. J. Litcofsky K. A. Ting C. Y. (2018). Electrophysiological and experimental-behavioral approaches to the study of intra-sentential code-switching. *Linguist. Approach. Bilingualism* 8 134–161. 10.1075/lab.16010.van

[B68] Van Hell J. G. Witteman M. J. (2009). “The neurocognition of switching between languages. A review of electrophysiological studies,” in *Multidisciplinary approaches to code switching*, eds Isurin L. Winford D. de Bot K. (Philadelphia: John Benjamins), 53–84. 10.1075/sibil.41.06hel

[B69] Van Petten C. Coulson S. Rubin S. Plante E. Parks M. (1999). Time course of word identification and semantic integration in spoken language. *J. Exp. Psychol.* 25 394–417. 10.1037/0278-7393.25.2.394 10093207

[B70] Van Petten C. Kutas M. (1991). Influences of semantic and syntactic context on open and closed class words. *Mem. Cogn.* 19 95–112. 10.3758/bf03198500 2017035

[B71] Van Petten C. Luka B. J. (2012). Prediction during language comprehension: Benefits, costs, and ERP components. *Int. J. Psychophysiol.* 83 176–190. 10.1016/j.ijpsycho.2011.09.015 22019481

[B72] Woolford E. (1983). Bilingual code-switching and syntactic theory. *Linguist. Inq.* 14 520–536.

[B73] Zeller J. P. Hentschel G. Ruigendijk E. (2016). “Psycholinguistic aspects of Belarusian-Russian language contact. An ERP study on code-switching between closely related languages,” in *Slavic Languages in Psycholinguistics. Chances and Challenges for Empirical and Experimental Research*, eds Anstatt T. Gattnar A. Clasmeier C. (Tübingen: Narr Francke Attempto), 257–278.

