# Supplementary material for: Code-Switching Does Not Equal Code-Switching. An Event-Related Potentials Study on Switching From L2 German to L1 Russian at Prepositions and Nouns
Source: Front Psychol. 2020 Jun 23;11:1387. doi: 10.3389/fpsyg.2020.01387 (PMC7324795; doi:10.3389/fpsyg.2020.01387)
Supplement: Supplementary file 1 [file Table_1.DOCX]

Supplementary Material

# Linear mixed-effects models reported in the main text

For all models reported in the study: *n* = 8902; Random factors: *Item* (*n* = 160); *Subject* (*n* = 31)

- *ant*: Anteriority (*posterior* = *post* versus *anterior = ant*)
- *cs*: Code-Switch (*no CS = no_cs* versus *CS = cs*)
- *point*: Word class/Switch point (*noun = n* vs. *preposition = p*)

## Early time window 1 (100–200 ms)

### Deviation coding

Formula: m100to200 ~ ant * cs * point + (1 + cs + point | subj) + (1 + cs * point | item)

***Random effects:***

| **Groups** | **Name** | **Variance** | **Std.Dev.** | **Corr** |  |  |
| --- | --- | --- | --- | --- | --- | --- |
| **item** | (Intercept) | 0.66 | 0.82 |  |  |  |
|  | cs | 2.39 | 1.55 | 0.17 |  |  |
|  | point | 3.72 | 1.93 | 0.03 | 0.04 |  |
|  | cs:point | 13.94 | 3.73 | -0.09 | -0.24 | -0.02 |
| **subj** | (Intercept) | 0.46 | 0.68 |  |  |  |
|  | cs | 0.73 | 0.86 | 0.43 |  |  |
|  | point | 1.81 | 1.34 | 0.62 | 0.23 |  |
| **Residual** |  | 39.43 | 6.28 |  |  |  |

***Fixed effects:***

| **Factor** | **Estimate** | **SE** | **df** | **t** | **p** |
| --- | --- | --- | --- | --- | --- |
| (Intercept) | -0.37 | 0.15 | 41.03 | -2.40 | 0.021 |
| ant | -0.25 | 0.13 | 8171.00 | -1.87 | 0.062 |
| cs | -0.11 | 0.24 | 47.22 | -0.44 | 0.661 |
| point | 1.33 | 0.32 | 48.14 | 4.20 | <0.001 |
| ant:cs | 0.30 | 0.27 | 8171.00 | 1.13 | 0.258 |
| ant:point | 0.65 | 0.27 | 8171.00 | 2.45 | 0.014 |
| cs:point | -0.97 | 0.40 | 157.50 | -2.44 | 0.016 |
| ant:cs:point | 0.01 | 0.53 | 8171.00 | 0.02 | 0.987 |

### Treatment coding, reference values: Anteriority = posterior, Point = P, CS = no CS

Formula: m100to200 ~ ant * point + cs * point + (1 + cs + point | subj) + (1 + cs * point | item)

***Random effects:***

| **Groups** | **Name** | **Variance** | **Std.Dev.** | **Corr** |  |  |
| --- | --- | --- | --- | --- | --- | --- |
| **item** | (Intercept) | 2.67 | 1.63 | 1.63 |  |  |
|  | cs = cs | 4.48 | 2.12 | -0.62 |  |  |
|  | point = n | 7.36 | 2.71 | -0.78 | 0.48 |  |
|  | cs = cs:point = n | 13.94 | 3.73 | 0.51 | -0.71 | -0.70 |
| **subj** | (Intercept) | 1.27 | 1.13 | 1.13 |  |  |
|  | cs = cs | 0.73 | 0.86 | 0.01 |  |  |
|  | point = n | 1.81 | 1.34 | -0.88 | -0.23 |  |
| **Residual** |  | 39.43 | 6.28 |  |  |  |

***Fixed effects:***

| **Factor** | **Estimate** | **SE** | **df** | **t** | **p** |
| --- | --- | --- | --- | --- | --- |
| (Intercept) | 0.55 | 0.29 | 64.93 | 1.90 | 0.061 |
| ant = ant | 0.08 | 0.19 | 8172.91 | 0.40 | 0.688 |
| point = n | -1.49 | 0.40 | 104.58 | -3.74 | <0.001 |
| cs = cs | -0.59 | 0.30 | 88.45 | -2.00 | 0.049 |
| ant = ant:point = n | -0.65 | 0.27 | 8172.91 | -2.44 | 0.015 |
| point = n:cs = cs | 0.97 | 0.40 | 157.56 | 2.44 | 0.016 |

### Treatment coding, reference values: Anteriority = posterior, Point = N, CS = no CS

Formula: m100to200 ~ ant * point + cs * point + (1 + cs + point | subj) + (1 + cs * point | item)

***Random effects:***

| **Groups** | **Name** | **Variance** | **Std.Dev.** | **Corr** |  |  |
| --- | --- | --- | --- | --- | --- | --- |
| **item** | (Intercept) | 3.10 | 1.76 |  |  |  |
|  | cs = cs | 7.27 | 2.70 | -0.71 |  |  |
|  | point = p | 7.36 | 2.71 | -0.82 | 0.60 |  |
|  | cs = cs:point = p | 13.94 | 3.73 | 0.61 | -0.83 | -0.70 |
| **subj** | (Intercept) | 0.42 | 0.65 |  |  |  |
|  | cs = cs | 0.73 | 0.86 | -0.46 |  |  |
|  | point = p | 1.81 | 1.34 | -0.55 | 0.23 |  |
| **Residual** |  | 39.43 | 6.28 |  |  |  |

***Fixed effects:***

| **Factor** | **Estimate** | **SE** | **df** | **t** | **p** |
| --- | --- | --- | --- | --- | --- |
| (Intercept) | -0.93 | 0.24 | 103.59 | -3.82 | <0.001 |
| ant = ant | -0.57 | 0.19 | 8172.91 | -3.05 | 0.002 |
| point = p | 1.49 | 0.40 | 104.59 | 3.74 | <0.001 |
| cs = cs | 0.38 | 0.32 | 106.11 | 1.18 | 0.243 |
| ant = ant:point = p | 0.65 | 0.27 | 8172.91 | 2.44 | 0.015 |
| point = p:cs = cs | -0.97 | 0.40 | 157.56 | -2.44 | 0.016 |

## Early time window 2 (200–500 ms)

### Deviation coding

Formula: m200to500 ~ ant * cs * point + (1 + cs * point | subj) + (1 + cs + point | item)

***Random effects:***

| **Groups** | **Name** | **Variance** | **Std.Dev.** | **Corr** |  |  |
| --- | --- | --- | --- | --- | --- | --- |
| **item** | (Intercept) | 0.44 | 0.66 |  |  |  |
|  | cs | 2.11 | 1.45 | 0.43 |  |  |
|  | point | 3.97 | 1.99 | -0.07 | -0.28 |  |
| **subj** | (Intercept) | 0.38 | 0.62 |  |  |  |
|  | cs | 0.94 | 0.97 | 0.38 |  |  |
|  | point | 2.31 | 1.52 | 0.33 | 0.47 |  |
|  | cs:point | 5.23 | 2.29 | 0.27 | -0.25 | -0.56 |
| **Residual** |  | 40.93 | 6.40 |  |  |  |

***Fixed effects:***

| **Factor** | **Estimate** | **SE** | **df** | **t** | **p** |
| --- | --- | --- | --- | --- | --- |
| (Intercept) | -0.58 | 0.14 | 38.06 | -4.12 | <0.001 |
| ant | -0.12 | 0.14 | 8308.82 | -0.89 | 0.372 |
| cs | -0.39 | 0.25 | 43.47 | -1.57 | 0.123 |
| point | 2.17 | 0.34 | 46.23 | 6.31 | <0.001 |
| ant:cs | 0.24 | 0.27 | 8308.82 | 0.90 | 0.371 |
| ant:point | 0.57 | 0.27 | 8308.82 | 2.09 | 0.037 |
| cs:point | -0.84 | 0.49 | 29.71 | -1.70 | 0.100 |
| ant:cs:point | -0.91 | 0.54 | 8308.82 | -1.68 | 0.093 |

### Treatment coding, reference values: Anteriority = anterior, Point = P, CS = no CS

Formula: m200to500 ~ ant * point * cs + (1 + cs * point | subj) + (1 + cs + point | item)

***Random effects:***

| **Groups** | **Name** | **Variance** | **Std.Dev.** | **Corr** |  |  |
| --- | --- | --- | --- | --- | --- | --- |
| **item** | (Intercept) | 1.86 | 1.37 |  |  |  |
|  | cs = cs | 2.11 | 1.45 | -0.53 |  |  |
|  | point = n | 3.97 | 1.99 | -0.85 | 0.28 |  |
| **subj** | (Intercept) | 1.41 | 1.19 |  |  |  |
|  | cs = cs | 1.70 | 1.30 | -0.36 |  |  |
|  | point = n | 5.55 | 2.36 | -0.86 | 0.43 |  |
|  | cs = cs:point = n | 5.23 | 2.29 | 0.59 | -0.69 | -0.84 |
| **Residual** |  | 40.93 | 6.40 |  |  |  |

***Fixed effects:***

| **Factor** | **Estimate** | **SE** | **df** | **t** | **p** |
| --- | --- | --- | --- | --- | --- |
| (Intercept) | 1.04 | 0.31 | 61.98 | 3.40 | 0.001 |
| ant = posterior | -0.27 | 0.27 | 8308.81 | -0.99 | 0.320 |
| point = n | -3.09 | 0.53 | 48.73 | -5.87 | <0.001 |
| cs = cs | -0.92 | 0.38 | 69.31 | -2.43 | 0.018 |
| ant = posterior:point = n | 1.02 | 0.38 | 8308.81 | 2.67 | 0.008 |
| ant = posterior:cs = cs | 0.21 | 0.38 | 8308.81 | 0.56 | 0.579 |
| point = n:cs = cs | 1.29 | 0.56 | 50.44 | 2.30 | 0.026 |
| ant = posterior:point = n:cs = cs | -0.91 | 0.54 | 8308.81 | -1.68 | 0.093 |

### Treatment coding, reference values: Anteriority = posterior, Point = P, CS = no CS

Formula: m200to500 ~ ant * point * cs + (1 + cs * point | subj) + (1 + cs + point | item)

***Random effects:***

| **Groups** | **Name** | **Variance** | **Std.Dev.** | **Corr** |  |  |
| --- | --- | --- | --- | --- | --- | --- |
| **item** | (Intercept) | 1.86 | 1.37 |  |  |  |
|  | cs = cs | 2.11 | 1.45 | -0.53 |  |  |
|  | point = n | 3.97 | 1.99 | -0.85 | 0.28 |  |
| **subj** | (Intercept) | 1.41 | 1.19 |  |  |  |
|  | cs = cs | 1.70 | 1.30 | -0.36 |  |  |
|  | point = n | 5.55 | 2.36 | -0.86 | 0.43 |  |
|  | cs = cs:point = n | 5.23 | 2.29 | 0.59 | -0.69 | -0.84 |
| **Residual** |  | 40.93 | 6.40 |  |  |  |

***Fixed effects:***

| **Factor** | **Estimate** | **SE** | **df** | **t** | **p** |
| --- | --- | --- | --- | --- | --- |
| (Intercept) | 0.77 | 0.31 | 61.98 | 2.53 | 0.014 |
| ant = anterior | 0.27 | 0.27 | 8308.81 | 0.99 | 0.320 |
| point = n | -2.07 | 0.53 | 48.73 | -3.94 | <0.001 |
| cs = cs | -0.70 | 0.38 | 69.31 | -1.87 | 0.066 |
| ant = anterior:point = n | -1.02 | 0.38 | 8308.81 | -2.67 | 0.008 |
| ant = anterior:cs = cs | -0.21 | 0.38 | 8308.81 | -0.56 | 0.579 |
| point = n:cs = cs | 0.38 | 0.56 | 50.44 | 0.68 | 0.501 |
| ant = anterior:point = n:cs = cs | 0.91 | 0.54 | 8308.81 | 1.68 | 0.093 |

### Treatment coding, reference values: Anteriority = anterior, Point = N, CS = CS

Formula: m200to500 ~ ant * point * cs + (1 + cs * point | subj) + (1 + cs + point | item)

***Random effects:***

| **Groups** | **Name** | **Variance** | **Std.Dev.** | **Corr** |  |  |
| --- | --- | --- | --- | --- | --- | --- |
| **item** | (Intercept) | 2.87 | 1.69 |  |  |  |
|  | cs = no_cs | 2.11 | 1.45 | -0.76 |  |  |
|  | point = p | 3.97 | 1.99 | -0.74 | 0.28 |  |
| **subj** | (Intercept) | 0.56 | 0.75 |  |  |  |
|  | cs = no_cs | 2.80 | 1.67 | -0.49 |  |  |
|  | point = p | 1.68 | 1.29 | -0.13 | -0.03 |  |
|  | cs = no_cs:point = p | 5.23 | 2.29 | 0.13 | -0.83 | -0.23 |
| **Residual** |  | 40.93 | 6.40 |  |  |  |

***Fixed effects:***

| **Factor** | **Estimate** | **SE** | **df** | **t** | **p** |
| --- | --- | --- | --- | --- | --- |
| (Intercept) | -1.68 | 0.27 | 98.92 | -6.22 | <0.001 |
| ant = posterior | 0.05 | 0.27 | 8308.81 | 0.20 | 0.840 |
| point = p | 1.80 | 0.39 | 79.79 | 4.60 | <0.001 |
| cs = no_cs | -0.38 | 0.42 | 56.31 | -0.89 | 0.376 |
| ant = posterior:point = p | -0.11 | 0.38 | 8308.81 | -0.29 | 0.775 |
| ant = posterior:cs = no_cs | 0.70 | 0.38 | 8308.81 | 1.82 | 0.069 |
| point = p:cs = no_cs | 1.29 | 0.56 | 50.42 | 2.30 | 0.026 |
| ant = posterior:point = p:cs = no_cs | -0.91 | 0.54 | 8308.81 | -1.68 | 0.093 |

### Treatment coding, reference values: Anteriority = posterior, Point = N, CS = CS

Formula: m200to500 ~ ant * point * cs + (1 + cs * point | subj) + (1 + cs + point | item)

***Random effects:***

| **Groups** | **Name** | **Variance** | **Std.Dev.** | **Corr** |  |  |
| --- | --- | --- | --- | --- | --- | --- |
| **item** | (Intercept) | 2.87 | 1.69 |  |  |  |
|  | cs = no_cs | 2.11 | 1.45 | -0.76 |  |  |
|  | point = p | 3.97 | 1.99 | -0.74 | 0.28 |  |
| **subj** | (Intercept) | 0.56 | 0.75 |  |  |  |
|  | cs = no_cs | 2.80 | 1.67 | -0.49 |  |  |
|  | point = p | 1.68 | 1.29 | -0.13 | -0.03 |  |
|  | cs = no_cs:point = p | 5.23 | 2.29 | 0.13 | -0.83 | -0.23 |
| **Residual** |  | 40.93 | 6.40 |  |  |  |

***Fixed effects:***

| **Factor** | **Estimate** | **SE** | **df** | **t** | **p** |
| --- | --- | --- | --- | --- | --- |
| (Intercept) | -1.62 | 0.27 | 98.91 | -6.01 | <0.001 |
| ant = anterior | -0.05 | 0.27 | 8308.82 | -0.20 | 0.840 |
| point = p | 1.69 | 0.39 | 79.79 | 4.32 | <0.001 |
| cs = no_cs | 0.32 | 0.42 | 56.31 | 0.77 | 0.447 |
| ant = anterior:point = p | 0.11 | 0.38 | 8308.82 | 0.29 | 0.775 |
| ant = anterior:cs = no_cs | -0.70 | 0.38 | 8308.82 | -1.82 | 0.069 |
| point = p:cs = no_cs | 0.38 | 0.56 | 50.42 | 0.68 | 0.501 |
| ant = anterior:point = p:cs = no_cs | 0.91 | 0.54 | 8308.82 | 1.68 | 0.093 |

## Late time window 1 (500–800 ms)

### Deviation coding

Formula: m500to800 ~ ant * cs * point + (1 + cs * point | subj) + (1 + cs + point | item)

***Random effects:***

| **Groups** | **Name** | **Variance** | **Std.Dev.** | **Corr** |  |  |
| --- | --- | --- | --- | --- | --- | --- |
| **item** | (Intercept) | 1.16 | 1.08 |  |  |  |
|  | cs | 3.96 | 1.99 | 0.23 |  |  |
|  | point | 3.36 | 1.83 | -0.06 | -0.33 |  |
| **subj** | (Intercept) | 1.45 | 1.20 |  |  |  |
|  | cs | 2.42 | 1.56 | 0.63 |  |  |
|  | point | 2.30 | 1.52 | -0.29 | -0.03 |  |
|  | cs:point | 6.87 | 2.62 | -0.09 | -0.47 | -0.49 |
| **Residual** |  | 53.38 | 7.31 |  |  |  |

***Fixed effects:***

| **Factor** | **Estimate** | **SE** | **df** | **t** | **p** |
| --- | --- | --- | --- | --- | --- |
| (Intercept) | -0.01 | 0.25 | 38.43 | -0.05 | 0.961 |
| ant | -1.46 | 0.15 | 8306.93 | -9.40 | <0.001 |
| cs | 1.00 | 0.36 | 42.95 | 2.81 | 0.007 |
| point | 0.40 | 0.35 | 42.62 | 1.17 | 0.249 |
| ant:cs | -1.20 | 0.31 | 8306.93 | -3.89 | <0.001 |
| ant:point | 0.93 | 0.31 | 8306.93 | 2.99 | 0.003 |
| cs:point | -1.56 | 0.56 | 29.58 | -2.76 | 0.010 |
| ant:cs:point | -0.12 | 0.62 | 8306.93 | -0.19 | 0.850 |

### Treatment coding, reference values: Anteriority = anterior, Point = P, CS = no CS

Formula: m500to800 ~ ant * point + cs * point + ant * cs + +(1 + cs * point | subj) + (1 + cs + point | item)

***Random effects:***

| **Groups** | **Name** | **Variance** | **Std.Dev.** | **Corr** |  |  |
| --- | --- | --- | --- | --- | --- | --- |
| **item** | (Intercept) | 2.99 | 1.73 |  |  |  |
|  | cs = cs | 3.96 | 1.99 | -0.61 |  |  |
|  | point = n | 3.36 | 1.83 | -0.69 | 0.33 |  |
| **subj** | (Intercept) | 1.53 | 1.24 |  |  |  |
|  | cs = cs | 2.22 | 1.49 | -0.32 |  |  |
|  | point = n | 5.97 | 2.44 | -0.71 | 0.50 |  |
|  | cs = cs:point = n | 6.87 | 2.62 | 0.63 | -0.39 | -0.84 |
| **Residual** |  | 53.38 | 7.31 |  |  |  |

***Fixed effects:***

| **Factor** | **Estimate** | **SE** | **df** | **t** | **p** |
| --- | --- | --- | --- | --- | --- |
| (Intercept) | -0.12 | 0.33 | 62.82 | -0.35 | 0.727 |
| ant = posterior | 0.39 | 0.27 | 8307.93 | 1.46 | 0.145 |
| point = n | -1.65 | 0.53 | 41.74 | -3.08 | 0.004 |
| cs = cs | -0.38 | 0.41 | 55.83 | -0.92 | 0.362 |
| ant = posterior:point = n | 0.93 | 0.31 | 8307.93 | 3.00 | 0.003 |
| point = n:cs = cs | 1.56 | 0.56 | 29.58 | 2.76 | 0.010 |
| ant = posterior:cs = cs | 1.20 | 0.31 | 8307.93 | 3.89 | <0.001 |

### Treatment coding, reference values: Anteriority = posterior, Point = P, CS = no CS

Formula: m500to800 ~ ant * point + cs * point + ant * cs + +(1 + cs * point | subj) + (1 + cs + point | item)

***Random effects:***

| **Groups** | **Name** | **Variance** | **Std.Dev.** | **Corr** |  |  |
| --- | --- | --- | --- | --- | --- | --- |
| **item** | (Intercept) | 2.99 | 1.73 |  |  |  |
|  | cs = cs | 3.96 | 1.99 | -0.61 |  |  |
|  | point = n | 3.36 | 1.83 | -0.69 | 0.33 |  |
| **subj** | (Intercept) | 1.53 | 1.24 |  |  |  |
|  | cs = cs | 2.22 | 1.49 | -0.32 |  |  |
|  | point = n | 5.97 | 2.44 | -0.71 | 0.50 |  |
|  | cs = cs:point = n | 6.87 | 2.62 | 0.63 | -0.39 | -0.84 |
| **Residual** |  | 53.38 | 7.31 |  |  |  |

***Fixed effects:***

| **Factor** | **Estimate** | **SE** | **df** | **t** | **p** |
| --- | --- | --- | --- | --- | --- |
| (Intercept) | 0.27 | 0.33 | 62.82 | 0.82 | 0.414 |
| ant = anterior | -0.39 | 0.27 | 8307.93 | -1.46 | 0.145 |
| point = n | -0.72 | 0.53 | 41.74 | -1.35 | 0.186 |
| cs = cs | 0.83 | 0.41 | 55.83 | 2.01 | 0.049 |
| ant = anterior:point = n | -0.93 | 0.31 | 8307.93 | -3.00 | 0.003 |
| point = n:cs = cs | 1.56 | 0.56 | 29.58 | 2.76 | 0.010 |
| ant = anterior:cs = cs | -1.20 | 0.31 | 8307.93 | -3.89 | <0.001 |

### Treatment coding, reference values: Anteriority = anterior, Point = N, CS = no CS

Formula: m500to800 ~ ant * point + cs * point + ant * cs + (1 + cs * point | subj) + (1 + cs + point | item)

***Random effects:***

| **Groups** | **Name** | **Variance** | **Std.Dev.** | **Corr** |  |  |
| --- | --- | --- | --- | --- | --- | --- |
| **item** | (Intercept) | 1.99 | 1.41 |  |  |  |
|  | cs = cs | 3.96 | 1.99 | -0.31 |  |  |
|  | point = p | 3.36 | 1.83 | -0.46 | -0.33 |  |
| **subj** | (Intercept) | 3.19 | 1.79 |  |  |  |
|  | cs = cs | 6.06 | 2.46 | -0.49 |  |  |
|  | point = p | 5.97 | 2.44 | -0.87 | 0.59 |  |
|  | cs = cs:point = p | 6.87 | 2.62 | 0.72 | -0.83 | -0.84 |
| **Residual** |  | 53.38 | 7.31 |  |  |  |

***Fixed effects:***

| **Factor** | **Estimate** | **SE** | **df** | **t** | **p** |
| --- | --- | --- | --- | --- | --- |
| (Intercept) | -1.76 | 0.40 | 45.57 | -4.44 | <0.001 |
| ant = posterior | 1.32 | 0.27 | 8307.93 | 4.90 | <0.001 |
| point = p | 1.65 | 0.53 | 41.75 | 3.08 | 0.004 |
| cs = cs | 1.18 | 0.54 | 42.27 | 2.18 | 0.035 |
| ant = posterior:point = p | -0.93 | 0.31 | 8307.93 | -3.00 | 0.003 |
| point = p:cs = cs | -1.56 | 0.56 | 29.59 | -2.76 | 0.010 |
| ant = posterior:cs = cs | 1.20 | 0.31 | 8307.93 | 3.89 | <0.001 |

### Treatment coding, reference values: Anteriority = posterior, Point = N, CS = no CS

Formula: m500to800 ~ ant * point + cs * point + ant * cs + (1 + cs * point | subj) + (1 + cs + point | item)

***Random effects:***

| **Groups** | **Name** | **Variance** | **Std.Dev.** | **Corr** |  |  |
| --- | --- | --- | --- | --- | --- | --- |
| **item** | (Intercept) | 1.99 | 1.41 |  |  |  |
|  | cs = cs | 3.96 | 1.99 | -0.31 |  |  |
|  | point = p | 3.36 | 1.83 | -0.46 | -0.33 |  |
| **subj** | (Intercept) | 3.19 | 1.79 |  |  |  |
|  | cs = cs | 6.06 | 2.46 | -0.49 |  |  |
|  | point = p | 5.97 | 2.44 | -0.87 | 0.59 |  |
|  | cs = cs:point = p | 6.87 | 2.62 | 0.72 | -0.83 | -0.84 |
| **Residual** |  | 53.38 | 7.31 |  |  |  |

***Fixed effects:***

| **Factor** | **Estimate** | **SE** | **df** | **t** | **p** |
| --- | --- | --- | --- | --- | --- |
| (Intercept) | -0.45 | 0.40 | 45.57 | -1.12 | 0.267 |
| ant = anterior | -1.32 | 0.27 | 8307.93 | -4.90 | <0.001 |
| point = p | 0.72 | 0.53 | 41.75 | 1.35 | 0.186 |
| cs = cs | 2.38 | 0.54 | 42.27 | 4.40 | <0.001 |
| ant = anterior:point = p | 0.93 | 0.31 | 8307.93 | 3.00 | 0.003 |
| point = p:cs = cs | -1.56 | 0.56 | 29.59 | -2.76 | 0.010 |
| ant = anterior:cs = cs | -1.20 | 0.31 | 8307.93 | -3.89 | <0.001 |

## Late time window 2 (800–1000 ms)

### Deviation coding

Formula: m800to1000 ~ ant * cs * point + (1 + cs * point | subj) + (1 + cs * point | item)

***Random effects:***

| **Groups** | **Name** | **Variance** | **Std.Dev.** | **Corr** |  |  |
| --- | --- | --- | --- | --- | --- | --- |
| **item** | (Intercept) | 1.45 | 1.21 |  |  |  |
|  | cs | 4.28 | 2.07 | 0.17 |  |  |
|  | point | 4.18 | 2.04 | 0.02 | -0.06 |  |
|  | cs:point | 16.13 | 4.02 | -0.21 | -0.16 | 0.34 |
| **subj** | (Intercept) | 2.67 | 1.63 |  |  |  |
|  | cs | 2.35 | 1.53 | 0.62 |  |  |
|  | point | 0.72 | 0.85 | -0.14 | 0.07 |  |
|  | cs:point | 0.62 | 0.78 | -0.23 | -0.43 | 0.01 |
| **Residual** |  | 60.33 | 7.77 |  |  |  |

***Fixed effects:***

| **Factor** | **Estimate** | **SE** | **df** | **t** | **p** |
| --- | --- | --- | --- | --- | --- |
| (Intercept) | 0.21 | 0.32 | 35.80 | 0.66 | 0.513 |
| ant | -1.44 | 0.16 | 8143.04 | -8.72 | <0.001 |
| cs | 1.66 | 0.36 | 44.01 | 4.60 | <0.001 |
| point | -0.72 | 0.28 | 56.67 | -2.60 | 0.012 |
| ant:cs | -1.07 | 0.33 | 8143.04 | -3.25 | 0.001 |
| ant:point | 0.72 | 0.33 | 8143.04 | 2.19 | 0.029 |
| cs:point | -0.07 | 0.48 | 60.44 | -0.15 | 0.878 |
| ant:cs:point | -0.35 | 0.66 | 8143.04 | -0.53 | 0.600 |

### Treatment coding, reference values: Anteriority = anterior, Point = N, CS = no CS

Formula: m800to1000 ~ ant * point + ant * cs + (1 + cs + point | subj) + (1 + cs + point | item)

***Random effects:***

| **Groups** | **Name** | **Variance** | **Std.Dev.** | **Corr** |  |
| --- | --- | --- | --- | --- | --- |
| **item** | (Intercept) | 2.83 | 1.68 |  |  |
|  | cs = cs | 4.21 | 2.05 | -0.43 |  |
|  | point = p | 4.07 | 2.02 | -0.53 | -0.08 |
| **subj** | (Intercept) | 2.10 | 1.45 |  |  |
|  | cs = cs | 2.32 | 1.52 | 0.16 |  |
|  | point = p | 0.71 | 0.84 | -0.49 | 0.05 |
| **Residual** |  | 61.41 | 7.84 |  |  |

***Fixed effects:***

| **Factor** | **Estimate** | **SE** | **df** | **t** | **p** |
| --- | --- | --- | --- | --- | --- |
| (Intercept) | -0.89 | 0.36 | 58.93 | -2.49 | 0.016 |
| ant = posterior | 1.26 | 0.29 | 8329.46 | 4.38 | <0.001 |
| point = p | -0.36 | 0.32 | 104.45 | -1.12 | 0.266 |
| cs = cs | 1.13 | 0.40 | 64.92 | 2.86 | 0.006 |
| ant = posterior:point = p | -0.72 | 0.33 | 8329.46 | -2.17 | 0.030 |
| ant = posterior:cs = cs | 1.07 | 0.33 | 8329.46 | 3.22 | 0.001 |

### Treatment coding, reference values: Anteriority = posterior, Point = N, CS = no CS

Formula: m800to1000 ~ ant * point + ant * cs + +(1 + cs + point | subj) + (1 + cs + point | item)

***Random effects:***

| **Groups** | **Name** | **Variance** | **Std.Dev.** | **Corr** |  |
| --- | --- | --- | --- | --- | --- |
| **item** | (Intercept) | 2.83 | 1.68 |  |  |
|  | cs = cs | 4.21 | 2.05 | -0.43 |  |
|  | point = p | 4.07 | 2.02 | -0.53 | -0.08 |
| **subj** | (Intercept) | 2.10 | 1.45 |  |  |
|  | cs = cs | 2.32 | 1.52 | 0.16 |  |
|  | point = p | 0.71 | 0.84 | -0.49 | 0.05 |
| **Residual** |  | 61.41 | 7.84 |  |  |

***Fixed effects:***

| **Factor** | **Estimate** | **SE** | **df** | **t** | **p** |
| --- | --- | --- | --- | --- | --- |
| (Intercept) | 0.37 | 0.36 | 58.93 | 1.04 | 0.303 |
| ant = anterior | -1.26 | 0.29 | 8329.46 | -4.38 | <0.001 |
| point = p | -1.08 | 0.32 | 104.45 | -3.36 | 0.001 |
| cs = cs | 2.20 | 0.40 | 64.92 | 5.56 | <0.001 |
| ant = anterior:point = p | 0.72 | 0.33 | 8329.46 | 2.17 | 0.030 |
| ant = anterior:cs = cs | -1.07 | 0.33 | 8329.46 | -3.22 | 0.001 |

# Sentence material

| ID | Quartet | Variant | List | Condition | Sentence context | P + Det | N | Sentence context + P + Det (Transl.) | N (Transl.) |
| --- | --- | --- | --- | --- | --- | --- | --- | --- | --- |
| 1 | 1 | a | 1 | No CS | Der Kapitän steuert das Schiff | in diesen | Hafen | The captain navigates the ship into this | harbor |
| 2 | 1 | a | 2 | Semantic | Der Kapitän steuert das Schiff | in diesen | Stall | The captain navigates the ship into this | barn |
| 3 | 1 | a | 3 | CS at N | Der Kapitän steuert das Schiff | in diesen | port | The captain navigates the ship into this | harbor |
| 4 | 1 | a | 4 | CS at P | Der Kapitän steuert das Schiff | v ėtot | port | The captain navigates the ship into this | harbor |
| 5 | 1 | b | 1 | Semantic | Der Matrose steuert das Boot | in diesen | Stall | The sailor navigates the boat into this | barn |
| 6 | 1 | b | 2 | CS at N | Der Matrose steuert das Boot | in diesen | port | The sailor navigates the boat into this | harbor |
| 7 | 1 | b | 3 | CS at P | Der Matrose steuert das Boot | v ėtot | port | The sailor navigates the boat into this | harbor |
| 8 | 1 | b | 4 | No CS | Der Matrose steuert das Boot | in diesen | Hafen | The sailor navigates the boat into this | harbor |
| 9 | 1 | c | 1 | CS at N | Der Lotse steuert den Dampfer | in diesen | port | The pilot navigates the steamboat into this | harbor |
| 10 | 1 | c | 2 | CS at P | Der Lotse steuert den Dampfer | v ėtot | port | The pilot navigates the steamboat into this | harbor |
| 11 | 1 | c | 3 | No CS | Der Lotse steuert den Dampfer | in diesen | Hafen | The pilot navigates the steamboat into this | harbor |
| 12 | 1 | c | 4 | Semantic | Der Lotse steuert den Dampfer | in diesen | Stall | The pilot navigates the steamboat into this | barn |
| 13 | 1 | d | 1 | CS at P | Der Steuermann steuert die Yacht | v ėtot | port | The cox navigates the yacht into this | harbor |
| 14 | 1 | d | 2 | No CS | Der Steuermann steuert die Yacht | in diesen | Hafen | The cox navigates the yacht into this | harbor |
| 15 | 1 | d | 3 | Semantic | Der Steuermann steuert die Yacht | in diesen | Stall | The cox navigates the yacht into this | barn |
| 16 | 1 | d | 4 | CS at N | Der Steuermann steuert die Yacht | in diesen | port | The cox navigates the yacht into this | harbor |
| 17 | 2 | a | 1 | No CS | Der Bergführer führt die Bergsteiger | auf diese | Berge | The mountain guide guides the alpinists onto these | mountains |
| 18 | 2 | a | 2 | Semantic | Der Bergführer führt die Bergsteiger | auf diese | Ordner | The mountain guide guides the alpinists onto these | folders |
| 19 | 2 | a | 3 | CS at N | Der Bergführer führt die Bergsteiger | auf diese | gory | The mountain guide guides the alpinists onto these | mountains |
| 20 | 2 | a | 4 | CS at P | Der Bergführer führt die Bergsteiger | na ėti | gory | The mountain guide guides the alpinists onto these | mountains |
| 21 | 2 | b | 1 | Semantic | Der Reiseleiter führt die Wanderer | auf diese | Ordner | The tourist guide guides the wanderer onto these | folders |
| 22 | 2 | b | 2 | CS at N | Der Reiseleiter führt die Wanderer | auf diese | gory | The tourist guide guides the wanderer onto these | mountains |
| 23 | 2 | b | 3 | CS at P | Der Reiseleiter führt die Wanderer | na ėti | gory | The tourist guide guides the wanderer onto these | mountains |
| 24 | 2 | b | 4 | No CS | Der Reiseleiter führt die Wanderer | auf diese | Berge | The tourist guide guides the wanderer onto these | mountains |
| 25 | 2 | c | 1 | CS at N | Der Einheimische führt die Kletterer | auf diese | gory | The local guides the mountain climbers onto these | mountains |
| 26 | 2 | c | 2 | CS at P | Der Einheimische führt die Kletterer | na ėti | gory | The local guides the mountain climbers onto these | mountains |
| 27 | 2 | c | 3 | No CS | Der Einheimische führt die Kletterer | auf diese | Berge | The local guides the mountain climbers onto these | mountains |
| 28 | 2 | c | 4 | Semantic | Der Einheimische führt die Kletterer | auf diese | Ordner | The local guides the mountain climbers onto these | folders |
| 29 | 2 | d | 1 | CS at P | Der Bergsteiger führt die Touristen | na ėti | gory | The alpinist guides the tourists onto these | mountains |
| 30 | 2 | d | 2 | No CS | Der Bergsteiger führt die Touristen | auf diese | Berge | The alpinist guides the tourists onto these | mountains |
| 31 | 2 | d | 3 | Semantic | Der Bergsteiger führt die Touristen | auf diese | Ordner | The alpinist guides the tourists onto these | folders |
| 32 | 2 | d | 4 | CS at N | Der Bergsteiger führt die Touristen | auf diese | gory | The alpinist guides the tourists onto these | mountains |
| 33 | 3 | a | 1 | No CS | Die Köchin tut das Gemüse | in diese | Töpfe | The cook puts the vegetables into these | pots |
| 34 | 3 | a | 2 | Semantic | Die Köchin tut das Gemüse | in diese | Berge | The cook puts the vegetables into these | mountains |
| 35 | 3 | a | 3 | CS at N | Die Köchin tut das Gemüse | in diese | kastrjuli | The cook puts the vegetables into these | pots |
| 36 | 3 | a | 4 | CS at P | Die Köchin tut das Gemüse | v ėti | kastrjuli | The cook puts the vegetables into these | pots |
| 37 | 3 | b | 1 | Semantic | Der Chefkoch tut die Nudeln | in diese | Berge | The chef puts the noodles into these | mountains |
| 38 | 3 | b | 2 | CS at N | Der Chefkoch tut die Nudeln | in diese | kastrjuli | The chef puts the noodles into these | pots |
| 39 | 3 | b | 3 | CS at P | Der Chefkoch tut die Nudeln | v ėti | kastrjuli | The chef puts the noodles into these | pots |
| 40 | 3 | b | 4 | No CS | Der Chefkoch tut die Nudeln | in diese | Töpfe | The chef puts the noodles into these | pots |
| 41 | 3 | c | 1 | CS at N | Die Mutter tut die Kartoffeln | in diese | kastrjuli | The mother puts the potatoes into these | pots |
| 42 | 3 | c | 2 | CS at P | Die Mutter tut die Kartoffeln | v ėti | kastrjuli | The mother puts the potatoes into these | pots |
| 43 | 3 | c | 3 | No CS | Die Mutter tut die Kartoffeln | in diese | Töpfe | The mother puts the potatoes into these | pots |
| 44 | 3 | c | 4 | Semantic | Die Mutter tut die Kartoffeln | in diese | Berge | The mother puts the potatoes into these | mountains |
| 45 | 3 | d | 1 | CS at P | Der Vater tut die Zutaten | v ėti | kastrjuli | The father puts the ingredients into these | pots |
| 46 | 3 | d | 2 | No CS | Der Vater tut die Zutaten | in diese | Töpfe | The father puts the ingredients into these | pots |
| 47 | 3 | d | 3 | Semantic | Der Vater tut die Zutaten | in diese | Berge | The father puts the ingredients into these | mountains |
| 48 | 3 | d | 4 | CS at N | Der Vater tut die Zutaten | in diese | kastrjuli | The father puts the ingredients into these | pots |
| 49 | 4 | a | 1 | No CS | Die Sekretärin heftet die Dokumente | in diese | Ordner | The secretary bastes the documents into these | folders |
| 50 | 4 | a | 2 | Semantic | Die Sekretärin heftet die Dokumente | in diese | Zimmer | The secretary bastes the documents into these | rooms |
| 51 | 4 | a | 3 | CS at N | Die Sekretärin heftet die Dokumente | in diese | papki | The secretary bastes the documents into these | folders |
| 52 | 4 | a | 4 | CS at P | Die Sekretärin heftet die Dokumente | v ėti | papki | The secretary bastes the documents into these | folders |
| 53 | 4 | b | 1 | Semantic | Der Beamte heftet die Unterlagen | in diese | Zimmer | The executive bastes the papers into these | rooms |
| 54 | 4 | b | 2 | CS at N | Der Beamte heftet die Unterlagen | in diese | papki | The executive bastes the papers into these | folders |
| 55 | 4 | b | 3 | CS at P | Der Beamte heftet die Unterlagen | v ėti | papki | The executive bastes the papers into these | folders |
| 56 | 4 | b | 4 | No CS | Der Beamte heftet die Unterlagen | in diese | Ordner | The executive bastes the papers into these | folders |
| 57 | 4 | c | 1 | CS at N | Der Bibliothekar heftet die Akten | in diese | papki | The librarian bastes the files into these | folders |
| 58 | 4 | c | 2 | CS at P | Der Bibliothekar heftet die Akten | v ėti | papki | The librarian bastes the files into these | folders |
| 59 | 4 | c | 3 | No CS | Der Bibliothekar heftet die Akten | in diese | Ordner | The librarian bastes the files into these | folders |
| 60 | 4 | c | 4 | Semantic | Der Bibliothekar heftet die Akten | in diese | Zimmer | The librarian bastes the files into these | rooms |
| 61 | 4 | d | 1 | CS at P | Der Anwalt heftet die Schriftstücke | v ėti | papki | The lawyer bastes the writs into these | folders |
| 62 | 4 | d | 2 | No CS | Der Anwalt heftet die Schriftstücke | in diese | Ordner | The lawyer bastes the writs into these | folders |
| 63 | 4 | d | 3 | Semantic | Der Anwalt heftet die Schriftstücke | in diese | Zimmer | The lawyer bastes the writs into these | rooms |
| 64 | 4 | d | 4 | CS at N | Der Anwalt heftet die Schriftstücke | in diese | papki | The lawyer bastes the writs into these | folders |
| 65 | 5 | a | 1 | No CS | Der Autofahrer lenkt den Wagen | in diese | Straße | The car driver navigates the car into this | street |
| 66 | 5 | a | 2 | Semantic | Der Autofahrer lenkt den Wagen | in diese | Flasche | The car driver navigates the car into this | bottle |
| 67 | 5 | a | 3 | CS at N | Der Autofahrer lenkt den Wagen | in diese | ulicu | The car driver navigates the car into this | street |
| 68 | 5 | a | 4 | CS at P | Der Autofahrer lenkt den Wagen | v ėtu | ulicu | The car driver navigates the car into this | street |
| 69 | 5 | b | 1 | Semantic | Der Busfahrer lenkt den Bus | in diese | Flasche | The bus driver navigates the bus into this | bottle |
| 70 | 5 | b | 2 | CS at N | Der Busfahrer lenkt den Bus | in diese | ulicu | The bus driver navigates the bus into this | street |
| 71 | 5 | b | 3 | CS at P | Der Busfahrer lenkt den Bus | v ėtu | ulicu | The bus driver navigates the bus into this | street |
| 72 | 5 | b | 4 | No CS | Der Busfahrer lenkt den Bus | in diese | Straße | The bus driver navigates the bus into this | street |
| 73 | 5 | c | 1 | CS at N | Der Taxifahrer lenkt das Taxi | in diese | ulicu | The taxi driver navigates the taxi into this | street |
| 74 | 5 | c | 2 | CS at P | Der Taxifahrer lenkt das Taxi | v ėtu | ulicu | The taxi driver navigates the taxi into this | street |
| 75 | 5 | c | 3 | No CS | Der Taxifahrer lenkt das Taxi | in diese | Straße | The taxi driver navigates the taxi into this | street |
| 76 | 5 | c | 4 | Semantic | Der Taxifahrer lenkt das Taxi | in diese | Flasche | The taxi driver navigates the taxi into this | bottle |
| 77 | 5 | d | 1 | CS at P | Der Chauffeur lenkt die Limousine | v ėtu | ulicu | The chauffeur navigates the limousine into this | street |
| 78 | 5 | d | 2 | No CS | Der Chauffeur lenkt die Limousine | in diese | Straße | The chauffeur navigates the limousine into this | street |
| 79 | 5 | d | 3 | Semantic | Der Chauffeur lenkt die Limousine | in diese | Flasche | The chauffeur navigates the limousine into this | bottle |
| 80 | 5 | d | 4 | CS at N | Der Chauffeur lenkt die Limousine | in diese | ulicu | The chauffeur navigates the limousine into this | street |
| 81 | 6 | a | 1 | No CS | Der Hirte treibt die Schafe | in diesen | Stall | The shepherd drives the sheep into this | barn |
| 82 | 6 | a | 2 | Semantic | Der Hirte treibt die Schafe | in diesen | Schrank | The shepherd drives the sheep into this | wardrobe |
| 83 | 6 | a | 3 | CS at N | Der Hirte treibt die Schafe | in diesen | chlev | The shepherd drives the sheep into this | barn |
| 84 | 6 | a | 4 | CS at P | Der Hirte treibt die Schafe | v ėtot | chlev | The shepherd drives the sheep into this | barn |
| 85 | 6 | b | 1 | Semantic | Der Bauer treibt die Kühe | in diesen | Schrank | The farmer drives the cows into this | wardrobe |
| 86 | 6 | b | 2 | CS at N | Der Bauer treibt die Kühe | in diesen | chlev | The farmer drives the cows into this | barn |
| 87 | 6 | b | 3 | CS at P | Der Bauer treibt die Kühe | v ėtot | chlev | The farmer drives the cows into this | barn |
| 88 | 6 | b | 4 | No CS | Der Bauer treibt die Kühe | in diesen | Stall | The farmer drives the cows into this | barn |
| 89 | 6 | c | 1 | CS at N | Der Knecht treibt die Schweine | in diesen | chlev | The farm hand drives the pigs into this | barn |
| 90 | 6 | c | 2 | CS at P | Der Knecht treibt die Schweine | v ėtot | chlev | The farm hand drives the pigs into this | barn |
| 91 | 6 | c | 3 | No CS | Der Knecht treibt die Schweine | in diesen | Stall | The farm hand drives the pigs into this | barn |
| 92 | 6 | c | 4 | Semantic | Der Knecht treibt die Schweine | in diesen | Schrank | The farm hand drives the pigs into this | wardrobe |
| 93 | 6 | d | 1 | CS at P | Die Magd treibt die Pferde | v ėtot | chlev | The maidservant drives the horses into this | barn |
| 94 | 6 | d | 2 | No CS | Die Magd treibt die Pferde | in diesen | Stall | The maidservant drives the horses into this | barn |
| 95 | 6 | d | 3 | Semantic | Die Magd treibt die Pferde | in diesen | Schrank | The maidservant drives the horses into this | wardrobe |
| 96 | 6 | d | 4 | CS at N | Die Magd treibt die Pferde | in diesen | chlev | The maidservant drives the horses into this | barn |
| 97 | 7 | a | 1 | No CS | Der Kellner stellt die Getränke | auf diesen | Tisch | The waiter places the drinks onto this | table |
| 98 | 7 | a | 2 | Semantic | Der Kellner stellt die Getränke | auf diesen | Koffer | The waiter places the drinks onto this | suitcase |
| 99 | 7 | a | 3 | CS at N | Der Kellner stellt die Getränke | auf diesen | stol | The waiter places the drinks onto this | table |
| 100 | 7 | a | 4 | CS at P | Der Kellner stellt die Getränke | na ėtot | stol | The waiter places the drinks onto this | table |
| 101 | 7 | b | 1 | Semantic | Der Koch stellt die Vorspeise | auf diesen | Koffer | The cook places the starters onto this | suitcase |
| 102 | 7 | b | 2 | CS at N | Der Koch stellt die Vorspeise | auf diesen | stol | The cook places the starters onto this | table |
| 103 | 7 | b | 3 | CS at P | Der Koch stellt die Vorspeise | na ėtot | stol | The cook places the starters onto this | table |
| 104 | 7 | b | 4 | No CS | Der Koch stellt die Vorspeise | auf diesen | Tisch | The cook places the starters onto this | table |
| 105 | 7 | c | 1 | CS at N | Der Gastgeber stellt den Kuchen | auf diesen | stol | The host places the cake onto this | table |
| 106 | 7 | c | 2 | CS at P | Der Gastgeber stellt den Kuchen | na ėtot | stol | The host places the cake onto this | table |
| 107 | 7 | c | 3 | No CS | Der Gastgeber stellt den Kuchen | auf diesen | Tisch | The host places the cake onto this | table |
| 108 | 7 | c | 4 | Semantic | Der Gastgeber stellt den Kuchen | auf diesen | Koffer | The host places the cake onto this | suitcase |
| 109 | 7 | d | 1 | CS at P | Der Wirt stellt das Essen | na ėtot | stol | The innkeeper places the dish onto this | table |
| 110 | 7 | d | 2 | No CS | Der Wirt stellt das Essen | auf diesen | Tisch | The innkeeper places the dish onto this | table |
| 111 | 7 | d | 3 | Semantic | Der Wirt stellt das Essen | auf diesen | Koffer | The innkeeper places the dish onto this | suitcase |
| 112 | 7 | d | 4 | CS at N | Der Wirt stellt das Essen | auf diesen | stol | The innkeeper places the dish onto this | table |
| 113 | 8 | a | 1 | No CS | Der Dompteur sperrt die Löwen | in diese | Käfige | The animal trainer shuts the lions into these | cages |
| 114 | 8 | a | 2 | Semantic | Der Dompteur sperrt die Löwen | in diese | Gläser | The animal trainer shuts the lions into these | glasses |
| 115 | 8 | a | 3 | CS at N | Der Dompteur sperrt die Löwen | in diese | kletki | The animal trainer shuts the lions into these | cages |
| 116 | 8 | a | 4 | CS at P | Der Dompteur sperrt die Löwen | v ėti | kletki | The animal trainer shuts the lions into these | cages |
| 117 | 8 | b | 1 | Semantic | Der Wärter sperrt die Tiger | in diese | Gläser | The zookeeper shuts the tigers into these | glasses |
| 118 | 8 | b | 2 | CS at N | Der Wärter sperrt die Tiger | in diese | kletki | The zookeeper shuts the tigers into these | cages |
| 119 | 8 | b | 3 | CS at P | Der Wärter sperrt die Tiger | v ėti | kletki | The zookeeper shuts the tigers into these | cages |
| 120 | 8 | b | 4 | No CS | Der Wärter sperrt die Tiger | in diese | Käfige | The zookeeper shuts the tigers into these | cages |
| 121 | 8 | c | 1 | CS at N | Der Tierarzt sperrt die Hunde | in diese | kletki | The veterinarian shuts the dogs into these | cages |
| 122 | 8 | c | 2 | CS at P | Der Tierarzt sperrt die Hunde | v ėti | kletki | The veterinarian shuts the dogs into these | cages |
| 123 | 8 | c | 3 | No CS | Der Tierarzt sperrt die Hunde | in diese | Käfige | The veterinarian shuts the dogs into these | cages |
| 124 | 8 | c | 4 | Semantic | Der Tierarzt sperrt die Hunde | in diese | Gläser | The veterinarian shuts the dogs into these | glasses |
| 125 | 8 | d | 1 | CS at P | Der Zirkusdirektor sperrt die Affen | v ėti | kletki | The ringmaster shuts the monkeys into these | cages |
| 126 | 8 | d | 2 | No CS | Der Zirkusdirektor sperrt die Affen | in diese | Käfige | The ringmaster shuts the monkeys into these | cages |
| 127 | 8 | d | 3 | Semantic | Der Zirkusdirektor sperrt die Affen | in diese | Gläser | The ringmaster shuts the monkeys into these | glasses |
| 128 | 8 | d | 4 | CS at N | Der Zirkusdirektor sperrt die Affen | in diese | kletki | The ringmaster shuts the monkeys into these | cages |
| 129 | 9 | a | 1 | No CS | Der Polizist sperrt die Verbrecher | in diese | Zelle | The police officer shuts the villains into this | cell |
| 130 | 9 | a | 2 | Semantic | Der Polizist sperrt die Verbrecher | in diese | Schüssel | The police officer shuts the villains into this | bowl |
| 131 | 9 | a | 3 | CS at N | Der Polizist sperrt die Verbrecher | in diese | kameru | The police officer shuts the villains into this | cell |
| 132 | 9 | a | 4 | CS at P | Der Polizist sperrt die Verbrecher | v ėtu | kameru | The police officer shuts the villains into this | cell |
| 133 | 9 | b | 1 | Semantic | Der Aufseher sperrt die Verurteilten | in diese | Schüssel | The keeper shuts the convicts into this | bowl |
| 134 | 9 | b | 2 | CS at N | Der Aufseher sperrt die Verurteilten | in diese | kameru | The keeper shuts the convicts into this | cell |
| 135 | 9 | b | 3 | CS at P | Der Aufseher sperrt die Verurteilten | v ėtu | kameru | The keeper shuts the convicts into this | cell |
| 136 | 9 | b | 4 | No CS | Der Aufseher sperrt die Verurteilten | in diese | Zelle | The keeper shuts the convicts into this | cell |
| 137 | 9 | c | 1 | CS at N | Der Kommissar sperrt die Kriminellen | in diese | kameru | The commissioner shuts the criminals into this | cell |
| 138 | 9 | c | 2 | CS at P | Der Kommissar sperrt die Kriminellen | v ėtu | kameru | The commissioner shuts the criminals into this | cell |
| 139 | 9 | c | 3 | No CS | Der Kommissar sperrt die Kriminellen | in diese | Zelle | The commissioner shuts the criminals into this | cell |
| 140 | 9 | c | 4 | Semantic | Der Kommissar sperrt die Kriminellen | in diese | Schüssel | The commissioner shuts the criminals into this | bowl |
| 141 | 9 | d | 1 | CS at P | Der Gefängniswärter sperrt die Diebe | v ėtu | kameru | The jailer shuts the thieves into this | cell |
| 142 | 9 | d | 2 | No CS | Der Gefängniswärter sperrt die Diebe | in diese | Zelle | The jailer shuts the thieves into this | cell |
| 143 | 9 | d | 3 | Semantic | Der Gefängniswärter sperrt die Diebe | in diese | Schüssel | The jailer shuts the thieves into this | bowl |
| 144 | 9 | d | 4 | CS at N | Der Gefängniswärter sperrt die Diebe | in diese | kameru | The jailer shuts the thieves into this | cell |
| 145 | 10 | a | 1 | No CS | Die Wirtin gießt das Bier | in diese | Gläser | The innkeeper pours the beer into these | glasses |
| 146 | 10 | a | 2 | Semantic | Die Wirtin gießt das Bier | in diese | Beete | The innkeeper pours the beer into these | beds |
| 147 | 10 | a | 3 | CS at N | Die Wirtin gießt das Bier | in diese | stakany | The innkeeper pours the beer into these | glasses |
| 148 | 10 | a | 4 | CS at P | Die Wirtin gießt das Bier | v ėti | stakany | The innkeeper pours the beer into these | glasses |
| 149 | 10 | b | 1 | Semantic | Die Kellnerin gießt den Wein | in diese | Beete | The waitress pours the wine into these | beds |
| 150 | 10 | b | 2 | CS at N | Die Kellnerin gießt den Wein | in diese | stakany | The waitress pours the wine into these | glasses |
| 151 | 10 | b | 3 | CS at P | Die Kellnerin gießt den Wein | v ėti | stakany | The waitress pours the wine into these | glasses |
| 152 | 10 | b | 4 | No CS | Die Kellnerin gießt den Wein | in diese | Gläser | The waitress pours the wine into these | glasses |
| 153 | 10 | c | 1 | CS at N | Die Gastgeberin gießt den Sekt | in diese | stakany | The hostess pours the sparkling wine into these | glasses |
| 154 | 10 | c | 2 | CS at P | Die Gastgeberin gießt den Sekt | v ėti | stakany | The hostess pours the sparkling wine into these | glasses |
| 155 | 10 | c | 3 | No CS | Die Gastgeberin gießt den Sekt | in diese | Gläser | The hostess pours the sparkling wine into these | glasses |
| 156 | 10 | c | 4 | Semantic | Die Gastgeberin gießt den Sekt | in diese | Beete | The hostess pours the sparkling wine into these | beds |
| 157 | 10 | d | 1 | CS at P | Der Barkeeper gießt das Getränk | v ėti | stakany | The barkeeper pours the drink into these | glasses |
| 158 | 10 | d | 2 | No CS | Der Barkeeper gießt das Getränk | in diese | Gläser | The barkeeper pours the drink into these | glasses |
| 159 | 10 | d | 3 | Semantic | Der Barkeeper gießt das Getränk | in diese | Beete | The barkeeper pours the drink into these | beds |
| 160 | 10 | d | 4 | CS at N | Der Barkeeper gießt das Getränk | in diese | stakany | The barkeeper pours the drink into these | glasses |
| 161 | 11 | a | 1 | No CS | Der Reisende packt das Gepäck | in diesen | Koffer | The traveler puts the luggage into this | suitcase |
| 162 | 11 | a | 2 | Semantic | Der Reisende packt das Gepäck | in diesen | Hafen | The traveler puts the luggage into this | harbor |
| 163 | 11 | a | 3 | CS at N | Der Reisende packt das Gepäck | in diesen | čemodan | The traveler puts the luggage into this | suitcase |
| 164 | 11 | a | 4 | CS at P | Der Reisende packt das Gepäck | v ėtot | čemodan | The traveler puts the luggage into this | suitcase |
| 165 | 11 | b | 1 | Semantic | Der Tourist packt die Unterwäsche | in diesen | Hafen | The tourist puts the underwear into this | harbor |
| 166 | 11 | b | 2 | CS at N | Der Tourist packt die Unterwäsche | in diesen | čemodan | The tourist puts the underwear into this | suitcase |
| 167 | 11 | b | 3 | CS at P | Der Tourist packt die Unterwäsche | v ėtot | čemodan | The tourist puts the underwear into this | suitcase |
| 168 | 11 | b | 4 | No CS | Der Tourist packt die Unterwäsche | in diesen | Koffer | The tourist puts the underwear into this | suitcase |
| 169 | 11 | c | 1 | CS at N | Der Urlauber packt die Socken | in diesen | čemodan | The vacationist puts the socks into this | suitcase |
| 170 | 11 | c | 2 | CS at P | Der Urlauber packt die Socken | v ėtot | čemodan | The vacationist puts the socks into this | suitcase |
| 171 | 11 | c | 3 | No CS | Der Urlauber packt die Socken | in diesen | Koffer | The vacationist puts the socks into this | suitcase |
| 172 | 11 | c | 4 | Semantic | Der Urlauber packt die Socken | in diesen | Hafen | The vacationist puts the socks into this | harbor |
| 173 | 11 | d | 1 | CS at P | Der Feriengast packt die Hemden | v ėtot | čemodan | The holiday guest puts the shirts into this | suitcase |
| 174 | 11 | d | 2 | No CS | Der Feriengast packt die Hemden | in diesen | Koffer | The holiday guest puts the shirts into this | suitcase |
| 175 | 11 | d | 3 | Semantic | Der Feriengast packt die Hemden | in diesen | Hafen | The holiday guest puts the shirts into this | harbor |
| 176 | 11 | d | 4 | CS at N | Der Feriengast packt die Hemden | in diesen | čemodan | The holiday guest puts the shirts into this | suitcase |
| 177 | 12 | a | 1 | No CS | Der Ladenbesitzer klebt die Plakate | auf diese | Wände | The shop owner pastes the bill onto these | walls |
| 178 | 12 | a | 2 | Semantic | Der Ladenbesitzer klebt die Plakate | auf diese | Töpfe | The shop owner pastes the bill onto these | pots |
| 179 | 12 | a | 3 | CS at N | Der Ladenbesitzer klebt die Plakate | auf diese | steny | The shop owner pastes the bill onto these | walls |
| 180 | 12 | a | 4 | CS at P | Der Ladenbesitzer klebt die Plakate | na ėti | steny | The shop owner pastes the bill onto these | walls |
| 181 | 12 | b | 1 | Semantic | Die Tochter klebt die Fotos | auf diese | Töpfe | The daughter pastes the photographs onto these | pots |
| 182 | 12 | b | 2 | CS at N | Die Tochter klebt die Fotos | auf diese | steny | The daughter pastes the photographs onto these | walls |
| 183 | 12 | b | 3 | CS at P | Die Tochter klebt die Fotos | na ėti | steny | The daughter pastes the photographs onto these | walls |
| 184 | 12 | b | 4 | No CS | Die Tochter klebt die Fotos | auf diese | Wände | The daughter pastes the photographs onto these | walls |
| 185 | 12 | c | 1 | CS at N | Der Sohn klebt die Poster | auf diese | steny | The son pastes the posters onto these | walls |
| 186 | 12 | c | 2 | CS at P | Der Sohn klebt die Poster | na ėti | steny | The son pastes the posters onto these | walls |
| 187 | 12 | c | 3 | No CS | Der Sohn klebt die Poster | auf diese | Wände | The son pastes the posters onto these | walls |
| 188 | 12 | c | 4 | Semantic | Der Sohn klebt die Poster | auf diese | Töpfe | The son pastes the posters onto these | pots |
| 189 | 12 | d | 1 | CS at P | Der Maler klebt die Tapeten | na ėti | steny | The painter pastes the wallpaper onto these | walls |
| 190 | 12 | d | 2 | No CS | Der Maler klebt die Tapeten | auf diese | Wände | The painter pastes the wallpaper onto these | walls |
| 191 | 12 | d | 3 | Semantic | Der Maler klebt die Tapeten | auf diese | Töpfe | The painter pastes the wallpaper onto these | pots |
| 192 | 12 | d | 4 | CS at N | Der Maler klebt die Tapeten | auf diese | steny | The painter pastes the wallpaper onto these | walls |
| 193 | 13 | a | 1 | No CS | Der Angestellte schreibt die Adresse | auf diesen | Umschlag | The employee writes the address onto this | envelope |
| 194 | 13 | a | 2 | Semantic | Der Angestellte schreibt die Adresse | auf diesen | Kuchen | The employee writes the address onto this | cake |
| 195 | 13 | a | 3 | CS at N | Der Angestellte schreibt die Adresse | auf diesen | konvert | The employee writes the address onto this | envelope |
| 196 | 13 | a | 4 | CS at P | Der Angestellte schreibt die Adresse | na ėtot | konvert | The employee writes the address onto this | envelope |
| 197 | 13 | b | 1 | Semantic | Die Großmutter schreibt die Anschrift | auf diesen | Kuchen | The grandmother writes the address onto this | cake |
| 198 | 13 | b | 2 | CS at N | Die Großmutter schreibt die Anschrift | auf diesen | konvert | The grandmother writes the address onto this | envelope |
| 199 | 13 | b | 3 | CS at P | Die Großmutter schreibt die Anschrift | na ėtot | konvert | The grandmother writes the address onto this | envelope |
| 200 | 13 | b | 4 | No CS | Die Großmutter schreibt die Anschrift | auf diesen | Umschlag | The grandmother writes the address onto this | envelope |
| 201 | 13 | c | 1 | CS at N | Der Postbote schreibt den Absender | auf diesen | konvert | The postman writes the sender address onto this | envelope |
| 202 | 13 | c | 2 | CS at P | Der Postbote schreibt den Absender | na ėtot | konvert | The postman writes the sender address onto this | envelope |
| 203 | 13 | c | 3 | No CS | Der Postbote schreibt den Absender | auf diesen | Umschlag | The postman writes the sender address onto this | envelope |
| 204 | 13 | c | 4 | Semantic | Der Postbote schreibt den Absender | auf diesen | Kuchen | The postman writes the sender address onto this | cake |
| 205 | 13 | d | 1 | CS at P | Der Absender schreibt den Namen | na ėtot | konvert | The sender writes the name onto this | envelope |
| 206 | 13 | d | 2 | No CS | Der Absender schreibt den Namen | auf diesen | Umschlag | The sender writes the name onto this | envelope |
| 207 | 13 | d | 3 | Semantic | Der Absender schreibt den Namen | auf diesen | Kuchen | The sender writes the name onto this | cake |
| 208 | 13 | d | 4 | CS at N | Der Absender schreibt den Namen | auf diesen | konvert | The sender writes the name onto this | envelope |
| 209 | 14 | a | 1 | No CS | Der Gärtner pflanzt die Kartoffeln | in diese | Beete | The gardener plants the potatoes into these | beds |
| 210 | 14 | a | 2 | Semantic | Der Gärtner pflanzt die Kartoffeln | in diese | Bäume | The gardener plants the potatoes into these | trees |
| 211 | 14 | a | 3 | CS at N | Der Gärtner pflanzt die Kartoffeln | in diese | grjadki | The gardener plants the potatoes into these | beds |
| 212 | 14 | a | 4 | CS at P | Der Gärtner pflanzt die Kartoffeln | v ėti | grjadki | The gardener plants the potatoes into these | beds |
| 213 | 14 | b | 1 | Semantic | Die Bauersfrau pflanzt die Gurken | in diese | Bäume | The farmer's wife plants the cucumbers into these | trees |
| 214 | 14 | b | 2 | CS at N | Die Bauersfrau pflanzt die Gurken | in diese | grjadki | The farmer's wife plants the cucumbers into these | beds |
| 215 | 14 | b | 3 | CS at P | Die Bauersfrau pflanzt die Gurken | v ėti | grjadki | The farmer's wife plants the cucumbers into these | beds |
| 216 | 14 | b | 4 | No CS | Die Bauersfrau pflanzt die Gurken | in diese | Beete | The farmer's wife plants the cucumbers into these | beds |
| 217 | 14 | c | 1 | CS at N | Der Landwirt pflanzt den Salat | in diese | grjadki | The farmer plants the salad into these | beds |
| 218 | 14 | c | 2 | CS at P | Der Landwirt pflanzt den Salat | v ėti | grjadki | The farmer plants the salad into these | beds |
| 219 | 14 | c | 3 | No CS | Der Landwirt pflanzt den Salat | in diese | Beete | The farmer plants the salad into these | beds |
| 220 | 14 | c | 4 | Semantic | Der Landwirt pflanzt den Salat | in diese | Bäume | The farmer plants the salad into these | trees |
| 221 | 14 | d | 1 | CS at P | Der Siedler pflanzt die Tomaten | v ėti | grjadki | The settler plants the tomatoes into these | beds |
| 222 | 14 | d | 2 | No CS | Der Siedler pflanzt die Tomaten | in diese | Beete | The settler plants the tomatoes into these | beds |
| 223 | 14 | d | 3 | Semantic | Der Siedler pflanzt die Tomaten | in diese | Bäume | The settler plants the tomatoes into these | trees |
| 224 | 14 | d | 4 | CS at N | Der Siedler pflanzt die Tomaten | in diese | grjadki | The settler plants the tomatoes into these | beds |
| 225 | 15 | a | 1 | No CS | Der Sekretär steckt den Brief | in diesen | Briefkasten | The secretary puts the letter into this | letter box |
| 226 | 15 | a | 2 | Semantic | Der Sekretär steckt den Brief | in diesen | Lastwagen | The secretary puts the letter into this | truck |
| 227 | 15 | a | 3 | CS at N | Der Sekretär steckt den Brief | in diesen | jaščik | The secretary puts the letter into this | letter box |
| 228 | 15 | a | 4 | CS at P | Der Sekretär steckt den Brief | v ėtot | jaščik | The secretary puts the letter into this | letter box |
| 229 | 15 | b | 1 | Semantic | Der Großvater steckt die Postkarte | in diesen | Lastwagen | The grandfather puts the postcard into this | truck |
| 230 | 15 | b | 2 | CS at N | Der Großvater steckt die Postkarte | in diesen | jaščik | The grandfather puts the postcard into this | letter box |
| 231 | 15 | b | 3 | CS at P | Der Großvater steckt die Postkarte | v ėtot | jaščik | The grandfather puts the postcard into this | letter box |
| 232 | 15 | b | 4 | No CS | Der Großvater steckt die Postkarte | in diesen | Briefkasten | The grandfather puts the postcard into this | letter box |
| 233 | 15 | c | 1 | CS at N | Die Professorin steckt den Eilbrief | in diesen | jaščik | The professor puts the dispatch into this | letter box |
| 234 | 15 | c | 2 | CS at P | Die Professorin steckt den Eilbrief | v ėtot | jaščik | The professor puts the dispatch into this | letter box |
| 235 | 15 | c | 3 | No CS | Die Professorin steckt den Eilbrief | in diesen | Briefkasten | The professor puts the dispatch into this | letter box |
| 236 | 15 | c | 4 | Semantic | Die Professorin steckt den Eilbrief | in diesen | Lastwagen | The professor puts the dispatch into this | truck |
| 237 | 15 | d | 1 | CS at P | Die Großmutter steckt das Päckchen | v ėtot | jaščik | The grandmother puts the parcel into this | letter box |
| 238 | 15 | d | 2 | No CS | Die Großmutter steckt das Päckchen | in diesen | Briefkasten | The grandmother puts the parcel into this | letter box |
| 239 | 15 | d | 3 | Semantic | Die Großmutter steckt das Päckchen | in diesen | Lastwagen | The grandmother puts the parcel into this | truck |
| 240 | 15 | d | 4 | CS at N | Die Großmutter steckt das Päckchen | in diesen | jaščik | The grandmother puts the parcel into this | letter box |
| 241 | 16 | a | 1 | No CS | Die Küchenhilfe wirft den Abfall | in diese | Eimer | The kitchen help throws the garbage into these | buckets |
| 242 | 16 | a | 2 | Semantic | Die Küchenhilfe wirft den Abfall | in diese | Schiffe | The kitchen help throws the garbage into these | ships |
| 243 | 16 | a | 3 | CS at N | Die Küchenhilfe wirft den Abfall | in diese | vëdra | The kitchen help throws the garbage into these | buckets |
| 244 | 16 | a | 4 | CS at P | Die Küchenhilfe wirft den Abfall | v ėti | vëdra | The kitchen help throws the garbage into these | buckets |
| 245 | 16 | b | 1 | Semantic | Die Haushaltshilfe wirft den Müll | in diese | Schiffe | The cleaner throws the waste into these | ships |
| 246 | 16 | b | 2 | CS at N | Die Haushaltshilfe wirft den Müll | in diese | vëdra | The cleaner throws the waste into these | buckets |
| 247 | 16 | b | 3 | CS at P | Die Haushaltshilfe wirft den Müll | v ėti | vëdra | The cleaner throws the waste into these | buckets |
| 248 | 16 | b | 4 | No CS | Die Haushaltshilfe wirft den Müll | in diese | Eimer | The cleaner throws the waste into these | buckets |
| 249 | 16 | c | 1 | CS at N | Der Koch wirft die Reste | in diese | vëdra | The cook throws the leftovers into these | buckets |
| 250 | 16 | c | 2 | CS at P | Der Koch wirft die Reste | v ėti | vëdra | The cook throws the leftovers into these | buckets |
| 251 | 16 | c | 3 | No CS | Der Koch wirft die Reste | in diese | Eimer | The cook throws the leftovers into these | buckets |
| 252 | 16 | c | 4 | Semantic | Der Koch wirft die Reste | in diese | Schiffe | The cook throws the leftovers into these | ships |
| 253 | 16 | d | 1 | CS at P | Der Jugendliche wirft die Dosen | v ėti | vëdra | The teenager throws the tins into these | buckets |
| 254 | 16 | d | 2 | No CS | Der Jugendliche wirft die Dosen | in diese | Eimer | The teenager throws the tins into these | buckets |
| 255 | 16 | d | 3 | Semantic | Der Jugendliche wirft die Dosen | in diese | Schiffe | The teenager throws the tins into these | ships |
| 256 | 16 | d | 4 | CS at N | Der Jugendliche wirft die Dosen | in diese | vëdra | The teenager throws the tins into these | buckets |
| 257 | 17 | a | 1 | No CS | Die Tante gießt den Tee | in diese | Tasse | The aunt pours the tea into this | cup |
| 258 | 17 | a | 2 | Semantic | Die Tante gießt den Tee | in diese | Zelle | The aunt pours the tea into this | cell |
| 259 | 17 | a | 3 | CS at N | Die Tante gießt den Tee | in diese | čašku | The aunt pours the tea into this | cup |
| 260 | 17 | a | 4 | CS at P | Die Tante gießt den Tee | v ėtu | čašku | The aunt pours the tea into this | cup |
| 261 | 17 | b | 1 | Semantic | Die Oma gießt den Kaffee | in diese | Zelle | The grandma pours the coffee into this | cell |
| 262 | 17 | b | 2 | CS at N | Die Oma gießt den Kaffee | in diese | čašku | The grandma pours the coffee into this | cup |
| 263 | 17 | b | 3 | CS at P | Die Oma gießt den Kaffee | v ėtu | čašku | The grandma pours the coffee into this | cup |
| 264 | 17 | b | 4 | No CS | Die Oma gießt den Kaffee | in diese | Tasse | The grandma pours the coffee into this | cup |
| 265 | 17 | c | 1 | CS at N | Der Opa gießt den Kakao | in diese | čašku | The grandpa pours the cocoa into this | cup |
| 266 | 17 | c | 2 | CS at P | Der Opa gießt den Kakao | v ėtu | čašku | The grandpa pours the cocoa into this | cup |
| 267 | 17 | c | 3 | No CS | Der Opa gießt den Kakao | in diese | Tasse | The grandpa pours the cocoa into this | cup |
| 268 | 17 | c | 4 | Semantic | Der Opa gießt den Kakao | in diese | Zelle | The grandpa pours the cocoa into this | cell |
| 269 | 17 | d | 1 | CS at P | Der Onkel gießt den Glühwein | v ėtu | čašku | The uncle pours the hot punch into this | cup |
| 270 | 17 | d | 2 | No CS | Der Onkel gießt den Glühwein | in diese | Tasse | The uncle pours the hot punch into this | cup |
| 271 | 17 | d | 3 | Semantic | Der Onkel gießt den Glühwein | in diese | Zelle | The uncle pours the hot punch into this | cell |
| 272 | 17 | d | 4 | CS at N | Der Onkel gießt den Glühwein | in diese | čašku | The uncle pours the hot punch into this | cup |
| 273 | 18 | a | 1 | No CS | Der Mann schüttet die Erdnüsse | in diese | Schüssel | The man pours the peanuts into this | bowl |
| 274 | 18 | a | 2 | Semantic | Der Mann schüttet die Erdnüsse | in diese | Straße | The man pours the peanuts into this | street |
| 275 | 18 | a | 3 | CS at N | Der Mann schüttet die Erdnüsse | in diese | misku | The man pours the peanuts into this | bowl |
| 276 | 18 | a | 4 | CS at P | Der Mann schüttet die Erdnüsse | v ėtu | misku | The man pours the peanuts into this | bowl |
| 277 | 18 | b | 1 | Semantic | Der Student schüttet das Müsli | in diese | Straße | The student pours the muesli into this | street |
| 278 | 18 | b | 2 | CS at N | Der Student schüttet das Müsli | in diese | misku | The student pours the muesli into this | bowl |
| 279 | 18 | b | 3 | CS at P | Der Student schüttet das Müsli | v ėtu | misku | The student pours the muesli into this | bowl |
| 280 | 18 | b | 4 | No CS | Der Student schüttet das Müsli | in diese | Schüssel | The student pours the muesli into this | bowl |
| 281 | 18 | c | 1 | CS at N | Das Kind schüttet die Haferflocken | in diese | misku | The child pours the oat flakes into this | bowl |
| 282 | 18 | c | 2 | CS at P | Das Kind schüttet die Haferflocken | v ėtu | misku | The child pours the oat flakes into this | bowl |
| 283 | 18 | c | 3 | No CS | Das Kind schüttet die Haferflocken | in diese | Schüssel | The child pours the oat flakes into this | bowl |
| 284 | 18 | c | 4 | Semantic | Das Kind schüttet die Haferflocken | in diese | Straße | The child pours the oat flakes into this | street |
| 285 | 18 | d | 1 | CS at P | Die Frau schüttet die Nüsse | v ėtu | misku | The woman pours the nuts into this | bowl |
| 286 | 18 | d | 2 | No CS | Die Frau schüttet die Nüsse | in diese | Schüssel | The woman pours the nuts into this | bowl |
| 287 | 18 | d | 3 | Semantic | Die Frau schüttet die Nüsse | in diese | Straße | The woman pours the nuts into this | street |
| 288 | 18 | d | 4 | CS at N | Die Frau schüttet die Nüsse | in diese | misku | The woman pours the nuts into this | bowl |
| 289 | 19 | a | 1 | No CS | Der Sohn schmiert die Butter | auf diese | Brötchen | The son spreads the butter onto these | rolls |
| 290 | 19 | a | 2 | Semantic | Der Sohn schmiert die Butter | auf diese | Plätze | The son spreads the butter onto these | places |
| 291 | 19 | a | 3 | CS at N | Der Sohn schmiert die Butter | auf diese | buločki | The son spreads the butter onto these | rolls |
| 292 | 19 | a | 4 | CS at P | Der Sohn schmiert die Butter | na ėti | buločki | The son spreads the butter onto these | rolls |
| 293 | 19 | b | 1 | Semantic | Die Tochter schmiert die Marmelade | auf diese | Plätze | The daughter spreads the jam onto these | places |
| 294 | 19 | b | 2 | CS at N | Die Tochter schmiert die Marmelade | auf diese | buločki | The daughter spreads the jam onto these | rolls |
| 295 | 19 | b | 3 | CS at P | Die Tochter schmiert die Marmelade | na ėti | buločki | The daughter spreads the jam onto these | rolls |
| 296 | 19 | b | 4 | No CS | Die Tochter schmiert die Marmelade | auf diese | Brötchen | The daughter spreads the jam onto these | rolls |
| 297 | 19 | c | 1 | CS at N | Der Großvater schmiert den Honig | auf diese | buločki | The grandfather spreads the honey onto these | rolls |
| 298 | 19 | c | 2 | CS at P | Der Großvater schmiert den Honig | na ėti | buločki | The grandfather spreads the honey onto these | rolls |
| 299 | 19 | c | 3 | No CS | Der Großvater schmiert den Honig | auf diese | Brötchen | The grandfather spreads the honey onto these | rolls |
| 300 | 19 | c | 4 | Semantic | Der Großvater schmiert den Honig | auf diese | Plätze | The grandfather spreads the honey onto these | places |
| 301 | 19 | d | 1 | CS at P | Der Enkel schmiert die Margarine | na ėti | buločki | The grandson spreads the margarine onto these | rolls |
| 302 | 19 | d | 2 | No CS | Der Enkel schmiert die Margarine | auf diese | Brötchen | The grandson spreads the margarine onto these | rolls |
| 303 | 19 | d | 3 | Semantic | Der Enkel schmiert die Margarine | auf diese | Plätze | The grandson spreads the margarine onto these | places |
| 304 | 19 | d | 4 | CS at N | Der Enkel schmiert die Margarine | auf diese | buločki | The grandson spreads the margarine onto these | rolls |
| 305 | 20 | a | 1 | No CS | Der Freund stellt das Fahrrad | in diesen | Schuppen | The friend puts the bicycle into this | shed |
| 306 | 20 | a | 2 | Semantic | Der Freund stellt das Fahrrad | in diesen | Abfluss | The friend puts the bicycle into this | drain |
| 307 | 20 | a | 3 | CS at N | Der Freund stellt das Fahrrad | in diesen | saraj | The friend puts the bicycle into this | shed |
| 308 | 20 | a | 4 | CS at P | Der Freund stellt das Fahrrad | v ėtot | saraj | The friend puts the bicycle into this | shed |
| 309 | 20 | b | 1 | Semantic | Der Motorradfahrer stellt das Motorrad | in diesen | Abfluss | The biker puts the motorbike into this | drain |
| 310 | 20 | b | 2 | CS at N | Der Motorradfahrer stellt das Motorrad | in diesen | saraj | The biker puts the motorbike into this | shed |
| 311 | 20 | b | 3 | CS at P | Der Motorradfahrer stellt das Motorrad | v ėtot | saraj | The biker puts the motorbike into this | shed |
| 312 | 20 | b | 4 | No CS | Der Motorradfahrer stellt das Motorrad | in diesen | Schuppen | The biker puts the motorbike into this | shed |
| 313 | 20 | c | 1 | CS at N | Der Nachbar stellt den Rasenmäher | in diesen | saraj | The neighbor puts the lawn mower into this | shed |
| 314 | 20 | c | 2 | CS at P | Der Nachbar stellt den Rasenmäher | v ėtot | saraj | The neighbor puts the lawn mower into this | shed |
| 315 | 20 | c | 3 | No CS | Der Nachbar stellt den Rasenmäher | in diesen | Schuppen | The neighbor puts the lawn mower into this | shed |
| 316 | 20 | c | 4 | Semantic | Der Nachbar stellt den Rasenmäher | in diesen | Abfluss | The neighbor puts the lawn mower into this | drain |
| 317 | 20 | d | 1 | CS at P | Der Zimmermann stellt das Werkzeug | v ėtot | saraj | The carpenter puts the tool kit into this | shed |
| 318 | 20 | d | 2 | No CS | Der Zimmermann stellt das Werkzeug | in diesen | Schuppen | The carpenter puts the tool kit into this | shed |
| 319 | 20 | d | 3 | Semantic | Der Zimmermann stellt das Werkzeug | in diesen | Abfluss | The carpenter puts the tool kit into this | drain |
| 320 | 20 | d | 4 | CS at N | Der Zimmermann stellt das Werkzeug | in diesen | saraj | The carpenter puts the tool kit into this | shed |
| 321 | 21 | a | 1 | No CS | Der Förster führt die Schulklasse | in diesen | Wald | The forester leads the school class into this | forest |
| 322 | 21 | a | 2 | Semantic | Der Förster führt die Schulklasse | in diesen | Tisch | The forester leads the school class into this | table |
| 323 | 21 | a | 3 | CS at N | Der Förster führt die Schulklasse | in diesen | les | The forester leads the school class into this | forest |
| 324 | 21 | a | 4 | CS at P | Der Förster führt die Schulklasse | v ėtot | les | The forester leads the school class into this | forest |
| 325 | 21 | b | 1 | Semantic | Der Jagdhund führt die Jäger | in diesen | Tisch | The hound dog leads the hunter into this | table |
| 326 | 21 | b | 2 | CS at N | Der Jagdhund führt die Jäger | in diesen | les | The hound dog leads the hunter into this | forest |
| 327 | 21 | b | 3 | CS at P | Der Jagdhund führt die Jäger | v ėtot | les | The hound dog leads the hunter into this | forest |
| 328 | 21 | b | 4 | No CS | Der Jagdhund führt die Jäger | in diesen | Wald | The hound dog leads the hunter into this | forest |
| 329 | 21 | c | 1 | CS at N | Der Jäger führt die Pilzsammler | in diesen | les | The hunter leads the mushroom pickers into this | forest |
| 330 | 21 | c | 2 | CS at P | Der Jäger führt die Pilzsammler | v ėtot | les | The hunter leads the mushroom pickers into this | forest |
| 331 | 21 | c | 3 | No CS | Der Jäger führt die Pilzsammler | in diesen | Wald | The hunter leads the mushroom pickers into this | forest |
| 332 | 21 | c | 4 | Semantic | Der Jäger führt die Pilzsammler | in diesen | Tisch | The hunter leads the mushroom pickers into this | table |
| 333 | 21 | d | 1 | CS at P | Der Holzfäller führt die Arbeiter | v ėtot | les | The lumberjack leads the worker into this | forest |
| 334 | 21 | d | 2 | No CS | Der Holzfäller führt die Arbeiter | in diesen | Wald | The lumberjack leads the worker into this | forest |
| 335 | 21 | d | 3 | Semantic | Der Holzfäller führt die Arbeiter | in diesen | Tisch | The lumberjack leads the worker into this | table |
| 336 | 21 | d | 4 | CS at N | Der Holzfäller führt die Arbeiter | in diesen | les | The lumberjack leads the worker into this | forest |
| 337 | 22 | a | 1 | No CS | Der Hund jagt die Katzen | auf diese | Bäume | The dog chases the cats onto these | trees |
| 338 | 22 | a | 2 | Semantic | Der Hund jagt die Katzen | auf diese | Tüten | The dog chases the cats onto these | bags |
| 339 | 22 | a | 3 | CS at N | Der Hund jagt die Katzen | auf diese | derev’ja | The dog chases the cats onto these | trees |
| 340 | 22 | a | 4 | CS at P | Der Hund jagt die Katzen | na ėti | derev’ja | The dog chases the cats onto these | trees |
| 341 | 22 | b | 1 | Semantic | Der Tiger jagt die Affen | auf diese | Tüten | The tiger chases the monkeys onto these | bags |
| 342 | 22 | b | 2 | CS at N | Der Tiger jagt die Affen | auf diese | derev’ja | The tiger chases the monkeys onto these | trees |
| 343 | 22 | b | 3 | CS at P | Der Tiger jagt die Affen | na ėti | derev’ja | The tiger chases the monkeys onto these | trees |
| 344 | 22 | b | 4 | No CS | Der Tiger jagt die Affen | auf diese | Bäume | The tiger chases the monkeys onto these | trees |
| 345 | 22 | c | 1 | CS at N | Der Fuchs jagt die Eichhörnchen | auf diese | derev'ja | The fox chases the squirrels onto these | trees |
| 346 | 22 | c | 2 | CS at P | Der Fuchs jagt die Eichhörnchen | na ėti | derev'ja | The fox chases the squirrels onto these | trees |
| 347 | 22 | c | 3 | No CS | Der Fuchs jagt die Eichhörnchen | auf diese | Bäume | The fox chases the squirrels onto these | trees |
| 348 | 22 | c | 4 | Semantic | Der Fuchs jagt die Eichhörnchen | auf diese | Tüten | The fox chases the squirrels onto these | bags |
| 349 | 22 | d | 1 | CS at P | Der Löwe jagt die Schimpansen | na ėti | derev'ja | The lion chases the chimpanzees onto these | trees |
| 350 | 22 | d | 2 | No CS | Der Löwe jagt die Schimpansen | auf diese | Bäume | The lion chases the chimpanzees onto these | trees |
| 351 | 22 | d | 3 | Semantic | Der Löwe jagt die Schimpansen | auf diese | Tüten | The lion chases the chimpanzees onto these | bags |
| 352 | 22 | d | 4 | CS at N | Der Löwe jagt die Schimpansen | auf diese | derev'ja | The lion chases the chimpanzees onto these | trees |
| 353 | 23 | a | 1 | No CS | Der Straßenfeger fegt den Schmutz | in diese | Ecken | The street cleaner sweeps the dirt into these | corners |
| 354 | 23 | a | 2 | Semantic | Der Straßenfeger fegt den Schmutz | in diese | Tragen | The street cleaner sweeps the dirt into these | stretchers |
| 355 | 23 | a | 3 | CS at N | Der Straßenfeger fegt den Schmutz | in diese | ugly | The street cleaner sweeps the dirt into these | corners |
| 356 | 23 | a | 4 | CS at P | Der Straßenfeger fegt den Schmutz | v ėti | ugly | The street cleaner sweeps the dirt into these | corners |
| 357 | 23 | b | 1 | Semantic | Die Putzfrau fegt die Krümel | in diese | Tragen | The cleaning woman sweeps the crumbs into these | stretchers |
| 358 | 23 | b | 2 | CS at N | Die Putzfrau fegt die Krümel | in diese | ugly | The cleaning woman sweeps the crumbs into these | corners |
| 359 | 23 | b | 3 | CS at P | Die Putzfrau fegt die Krümel | v ėti | ugly | The cleaning woman sweeps the crumbs into these | corners |
| 360 | 23 | b | 4 | No CS | Die Putzfrau fegt die Krümel | in diese | Ecken | The cleaning woman sweeps the crumbs into these | corners |
| 361 | 23 | c | 1 | CS at N | Der Straßenkehrer fegt den Dreck | in diese | ugly | The road sweeper sweeps the soil into these | corners |
| 362 | 23 | c | 2 | CS at P | Der Straßenkehrer fegt den Dreck | v ėti | ugly | The road sweeper sweeps the soil into these | corners |
| 363 | 23 | c | 3 | No CS | Der Straßenkehrer fegt den Dreck | in diese | Ecken | The road sweeper sweeps the soil into these | corners |
| 364 | 23 | c | 4 | Semantic | Der Straßenkehrer fegt den Dreck | in diese | Tragen | The road sweeper sweeps the soil into these | stretchers |
| 365 | 23 | d | 1 | CS at P | Der Hausmeister fegt den Staub | v ėti | ugly | The caretaker sweeps the dust into these | corners |
| 366 | 23 | d | 2 | No CS | Der Hausmeister fegt den Staub | in diese | Ecken | The caretaker sweeps the dust into these | corners |
| 367 | 23 | d | 3 | Semantic | Der Hausmeister fegt den Staub | in diese | Tragen | The caretaker sweeps the dust into these | stretchers |
| 368 | 23 | d | 4 | CS at N | Der Hausmeister fegt den Staub | in diese | ugly | The caretaker sweeps the dust into these | corners |
| 369 | 24 | a | 1 | No CS | Der Vater bringt die Söhne | in diese | Schule | The father takes the sons into this | school |
| 370 | 24 | a | 2 | Semantic | Der Vater bringt die Söhne | in diese | Wunde | The father takes the sons into this | wound |
| 371 | 24 | a | 3 | CS at N | Der Vater bringt die Söhne | in diese | školu | The father takes the sons into this | school |
| 372 | 24 | a | 4 | CS at P | Der Vater bringt die Söhne | v ėtu | školu | The father takes the sons into this | school |
| 373 | 24 | b | 1 | Semantic | Die Mutter bringt die Töchter | in diese | Wunde | The mother takes the daughters into this | wound |
| 374 | 24 | b | 2 | CS at N | Die Mutter bringt die Töchter | in diese | školu | The mother takes the daughters into this | school |
| 375 | 24 | b | 3 | CS at P | Die Mutter bringt die Töchter | v ėtu | školu | The mother takes the daughters into this | school |
| 376 | 24 | b | 4 | No CS | Die Mutter bringt die Töchter | in diese | Schule | The mother takes the daughters into this | school |
| 377 | 24 | c | 1 | CS at N | Der Lehrer bringt die Kinder | in diese | školu | The teacher takes the children into this | school |
| 378 | 24 | c | 2 | CS at P | Der Lehrer bringt die Kinder | v ėtu | školu | The teacher takes the children into this | school |
| 379 | 24 | c | 3 | No CS | Der Lehrer bringt die Kinder | in diese | Schule | The teacher takes the children into this | school |
| 380 | 24 | c | 4 | Semantic | Der Lehrer bringt die Kinder | in diese | Wunde | The teacher takes the children into this | wound |
| 381 | 24 | d | 1 | CS at P | Der Busfahrer bringt die Schüler | v ėtu | školu | The bus driver takes the pupils into this | school |
| 382 | 24 | d | 2 | No CS | Der Busfahrer bringt die Schüler | in diese | Schule | The bus driver takes the pupils into this | school |
| 383 | 24 | d | 3 | Semantic | Der Busfahrer bringt die Schüler | in diese | Wunde | The bus driver takes the pupils into this | wound |
| 384 | 24 | d | 4 | CS at N | Der Busfahrer bringt die Schüler | in diese | školu | The bus driver takes the pupils into this | school |
| 385 | 25 | a | 1 | No CS | Der Platzanweiser bittet die Zuschauer | auf diese | Plätze | The usher asks the spectators onto these | places |
| 386 | 25 | a | 2 | Semantic | Der Platzanweiser bittet die Zuschauer | auf diese | Brötchen | The usher asks the spectators onto these | rolls |
| 387 | 25 | a | 3 | CS at N | Der Platzanweiser bittet die Zuschauer | auf diese | mesta | The usher asks the spectators onto these | places |
| 388 | 25 | a | 4 | CS at P | Der Platzanweiser bittet die Zuschauer | na ėti | mesta | The usher asks the spectators onto these | places |
| 389 | 25 | b | 1 | Semantic | Der Gastgeber bittet die Gäste | auf diese | Brötchen | The host asks the guests onto these | rolls |
| 390 | 25 | b | 2 | CS at N | Der Gastgeber bittet die Gäste | auf diese | mesta | The host asks the guests onto these | places |
| 391 | 25 | b | 3 | CS at P | Der Gastgeber bittet die Gäste | na ėti | mesta | The host asks the guests onto these | places |
| 392 | 25 | b | 4 | No CS | Der Gastgeber bittet die Gäste | auf diese | Plätze | The host asks the guests onto these | places |
| 393 | 25 | c | 1 | CS at N | Die Stewardess bittet die Fluggäste | auf diese | mesta | The stewardess asks the passengers onto these | places |
| 394 | 25 | c | 2 | CS at P | Die Stewardess bittet die Fluggäste | na ėti | mesta | The stewardess asks the passengers onto these | places |
| 395 | 25 | c | 3 | No CS | Die Stewardess bittet die Fluggäste | auf diese | Plätze | The stewardess asks the passengers onto these | places |
| 396 | 25 | c | 4 | Semantic | Die Stewardess bittet die Fluggäste | auf diese | Brötchen | The stewardess asks the passengers onto these | rolls |
| 397 | 25 | d | 1 | CS at P | Der Schaffner bittet die Passagiere | na ėti | mesta | The conductor asks the passengers onto these | places |
| 398 | 25 | d | 2 | No CS | Der Schaffner bittet die Passagiere | auf diese | Plätze | The conductor asks the passengers onto these | places |
| 399 | 25 | d | 3 | Semantic | Der Schaffner bittet die Passagiere | auf diese | Brötchen | The conductor asks the passengers onto these | rolls |
| 400 | 25 | d | 4 | CS at N | Der Schaffner bittet die Passagiere | auf diese | mesta | The conductor asks the passengers onto these | places |
| 401 | 26 | a | 1 | No CS | Der Verkäufer packt die Waren | in diese | Tüten | The sales person packs the goods into these | bags |
| 402 | 26 | a | 2 | Semantic | Der Verkäufer packt die Waren | in diese | Käfige | The sales person packs the goods into these | cages |
| 403 | 26 | a | 3 | CS at N | Der Verkäufer packt die Waren | in diese | pakety | The sales person packs the goods into these | bags |
| 404 | 26 | a | 4 | CS at P | Der Verkäufer packt die Waren | v ėti | pakety | The sales person packs the goods into these | bags |
| 405 | 26 | b | 1 | Semantic | Der Kunde packt die Einkäufe | in diese | Käfige | The customer packs the purchases into these | cages |
| 406 | 26 | b | 2 | CS at N | Der Kunde packt die Einkäufe | in diese | pakety | The customer packs the purchases into these | bags |
| 407 | 26 | b | 3 | CS at P | Der Kunde packt die Einkäufe | v ėti | pakety | The customer packs the purchases into these | bags |
| 408 | 26 | b | 4 | No CS | Der Kunde packt die Einkäufe | in diese | Tüten | The customer packs the purchases into these | bags |
| 409 | 26 | c | 1 | CS at N | Der Kassierer packt die Lebensmittel | in diese | pakety | The store cashier packs the groceries into these | bags |
| 410 | 26 | c | 2 | CS at P | Der Kassierer packt die Lebensmittel | v ėti | pakety | The store cashier packs the groceries into these | bags |
| 411 | 26 | c | 3 | No CS | Der Kassierer packt die Lebensmittel | in diese | Tüten | The store cashier packs the groceries into these | bags |
| 412 | 26 | c | 4 | Semantic | Der Kassierer packt die Lebensmittel | in diese | Käfige | The store cashier packs the groceries into these | cages |
| 413 | 26 | d | 1 | CS at P | Der Metzger packt die Würstchen | v ėti | pakety | The butcher packs the sausages into these | bags |
| 414 | 26 | d | 2 | No CS | Der Metzger packt die Würstchen | in diese | Tüten | The butcher packs the sausages into these | bags |
| 415 | 26 | d | 3 | Semantic | Der Metzger packt die Würstchen | in diese | Käfige | The butcher packs the sausages into these | cages |
| 416 | 26 | d | 4 | CS at N | Der Metzger packt die Würstchen | in diese | pakety | The butcher packs the sausages into these | bags |
| 417 | 27 | a | 1 | No CS | Die Arzthelferin führt die Patienten | in diese | Zimmer | The doctor's assistant leads the patients into these | rooms |
| 418 | 27 | a | 2 | Semantic | Die Arzthelferin führt die Patienten | in diese | Wände | The doctor's assistant leads the patients into these | walls |
| 419 | 27 | a | 3 | CS at N | Die Arzthelferin führt die Patienten | in diese | komnaty | The doctor's assistant leads the patients into these | rooms |
| 420 | 27 | a | 4 | CS at P | Die Arzthelferin führt die Patienten | v ėti | komnaty | The doctor's assistant leads the patients into these | rooms |
| 421 | 27 | b | 1 | Semantic | Die Sekretärin führt die Kunden | in diese | Wände | The secretary leads the customers into these | walls |
| 422 | 27 | b | 2 | CS at N | Die Sekretärin führt die Kunden | in diese | komnaty | The secretary leads the customers into these | rooms |
| 423 | 27 | b | 3 | CS at P | Die Sekretärin führt die Kunden | v ėti | komnaty | The secretary leads the customers into these | rooms |
| 424 | 27 | b | 4 | No CS | Die Sekretärin führt die Kunden | in diese | Zimmer | The secretary leads the customers into these | rooms |
| 425 | 27 | c | 1 | CS at N | Der Diener führt die Gäste | in diese | komnaty | The servant leads the guests into these | rooms |
| 426 | 27 | c | 2 | CS at P | Der Diener führt die Gäste | v ėti | komnaty | The servant leads the guests into these | rooms |
| 427 | 27 | c | 3 | No CS | Der Diener führt die Gäste | in diese | Zimmer | The servant leads the guests into these | rooms |
| 428 | 27 | c | 4 | Semantic | Der Diener führt die Gäste | in diese | Wände | The servant leads the guests into these | walls |
| 429 | 27 | d | 1 | CS at P | Der Assistent führt die Wartenden | v ėti | komnaty | The assistant leads the waiting people into these | rooms |
| 430 | 27 | d | 2 | No CS | Der Assistent führt die Wartenden | in diese | Zimmer | The assistant leads the waiting people into these | rooms |
| 431 | 27 | d | 3 | Semantic | Der Assistent führt die Wartenden | in diese | Wände | The assistant leads the waiting people into these | walls |
| 432 | 27 | d | 4 | CS at N | Der Assistent führt die Wartenden | in diese | komnaty | The assistant leads the waiting people into these | rooms |
| 433 | 28 | a | 1 | No CS | Die Matrosen laden den Proviant | auf diese | Schiffe | The sailors load the supplies onto these | ships |
| 434 | 28 | a | 2 | Semantic | Die Matrosen laden den Proviant | auf diese | Ecken | The sailors load the supplies onto these | corners |
| 435 | 28 | a | 3 | CS at N | Die Matrosen laden den Proviant | auf diese | korabli | The sailors load the supplies onto these | ships |
| 436 | 28 | a | 4 | CS at P | Die Matrosen laden den Proviant | na ėti | korabli | The sailors load the supplies onto these | ships |
| 437 | 28 | b | 1 | Semantic | Die Seeräuber laden die Beute | auf diese | Ecken | The pirates load the haul onto these | corners |
| 438 | 28 | b | 2 | CS at N | Die Seeräuber laden die Beute | auf diese | korabli | The pirates load the haul onto these | ships |
| 439 | 28 | b | 3 | CS at P | Die Seeräuber laden die Beute | na ėti | korabli | The pirates load the haul onto these | ships |
| 440 | 28 | b | 4 | No CS | Die Seeräuber laden die Beute | auf diese | Schiffe | The pirates load the haul onto these | ships |
| 441 | 28 | c | 1 | CS at N | Die Hafenarbeiter laden die Container | auf diese | korabli | The dock workers load the containers onto these | ships |
| 442 | 28 | c | 2 | CS at P | Die Hafenarbeiter laden die Container | na ėti | korabli | The dock workers load the containers onto these | ships |
| 443 | 28 | c | 3 | No CS | Die Hafenarbeiter laden die Container | auf diese | Schiffe | The dock workers load the containers onto these | ships |
| 444 | 28 | c | 4 | Semantic | Die Hafenarbeiter laden die Container | auf diese | Ecken | The dock workers load the containers onto these | corners |
| 445 | 28 | d | 1 | CS at P | Die Seeleute laden die Vorräte | na ėti | korabli | The seamen load the provisions onto these | ships |
| 446 | 28 | d | 2 | No CS | Die Seeleute laden die Vorräte | auf diese | Schiffe | The seamen load the provisions onto these | ships |
| 447 | 28 | d | 3 | Semantic | Die Seeleute laden die Vorräte | auf diese | Ecken | The seamen load the provisions onto these | corners |
| 448 | 28 | d | 4 | CS at N | Die Seeleute laden die Vorräte | auf diese | korabli | The seamen load the provisions onto these | ships |
| 449 | 29 | a | 1 | No CS | Der Winzer füllt den Wein | in diese | Flasche | The winegrower fills the wine into this | bottle |
| 450 | 29 | a | 2 | Semantic | Der Winzer füllt den Wein | in diese | Schule | The winegrower fills the wine into this | school |
| 451 | 29 | a | 3 | CS at N | Der Winzer füllt den Wein | in diese | butylku | The winegrower fills the wine into this | bottle |
| 452 | 29 | a | 4 | CS at P | Der Winzer füllt den Wein | v ėtu | butylku | The winegrower fills the wine into this | bottle |
| 453 | 29 | b | 1 | Semantic | Der Bierbrauer füllt das Bier | in diese | Schule | The brewer fills the beer into this | school |
| 454 | 29 | b | 2 | CS at N | Der Bierbrauer füllt das Bier | in diese | butylku | The brewer fills the beer into this | bottle |
| 455 | 29 | b | 3 | CS at P | Der Bierbrauer füllt das Bier | v ėtu | butylku | The brewer fills the beer into this | bottle |
| 456 | 29 | b | 4 | No CS | Der Bierbrauer füllt das Bier | in diese | Flasche | The brewer fills the beer into this | bottle |
| 457 | 29 | c | 1 | CS at N | Der Wanderer füllt das Wasser | in diese | butylku | The wanderer fills the water into this | bottle |
| 458 | 29 | c | 2 | CS at P | Der Wanderer füllt das Wasser | v ėtu | butylku | The wanderer fills the water into this | bottle |
| 459 | 29 | c | 3 | No CS | Der Wanderer füllt das Wasser | in diese | Flasche | The wanderer fills the water into this | bottle |
| 460 | 29 | c | 4 | Semantic | Der Wanderer füllt das Wasser | in diese | Schule | The wanderer fills the water into this | school |
| 461 | 29 | d | 1 | CS at P | Der Bauer füllt die Milch | v ėtu | butylku | The farmer fills the milk into this | bottle |
| 462 | 29 | d | 2 | No CS | Der Bauer füllt die Milch | in diese | Flasche | The farmer fills the milk into this | bottle |
| 463 | 29 | d | 3 | Semantic | Der Bauer füllt die Milch | in diese | Schule | The farmer fills the milk into this | school |
| 464 | 29 | d | 4 | CS at N | Der Bauer füllt die Milch | in diese | butylku | The farmer fills the milk into this | bottle |
| 465 | 30 | a | 1 | No CS | Der Ermittler lockt den Räuber | in diese | Falle | The investigator lures the robber into this | trap |
| 466 | 30 | a | 2 | Semantic | Der Ermittler lockt den Räuber | in diese | Spüle | The investigator lures the robber into this | sink |
| 467 | 30 | a | 3 | CS at N | Der Ermittler lockt den Räuber | in diese | lovušku | The investigator lures the robber into this | trap |
| 468 | 30 | a | 4 | CS at P | Der Ermittler lockt den Räuber | v ėtu | lovušku | The investigator lures the robber into this | trap |
| 469 | 30 | b | 1 | Semantic | Der Detektiv lockt den Dieb | in diese | Spüle | The detective lures the thief into this | sink |
| 470 | 30 | b | 2 | CS at N | Der Detektiv lockt den Dieb | in diese | lovušku | The detective lures the thief into this | trap |
| 471 | 30 | b | 3 | CS at P | Der Detektiv lockt den Dieb | v ėtu | lovušku | The detective lures the thief into this | trap |
| 472 | 30 | b | 4 | No CS | Der Detektiv lockt den Dieb | in diese | Falle | The detective lures the thief into this | trap |
| 473 | 30 | c | 1 | CS at N | Der Polizist lockt den Verbrecher | in diese | lovušku | The policeman lures the criminal into this | trap |
| 474 | 30 | c | 2 | CS at P | Der Polizist lockt den Verbrecher | v ėtu | lovušku | The policeman lures the criminal into this | trap |
| 475 | 30 | c | 3 | No CS | Der Polizist lockt den Verbrecher | in diese | Falle | The policeman lures the criminal into this | trap |
| 476 | 30 | c | 4 | Semantic | Der Polizist lockt den Verbrecher | in diese | Spüle | The policeman lures the criminal into this | sink |
| 477 | 30 | d | 1 | CS at P | Der Kommissar lockt den Mörder | v ėtu | lovušku | The commissioner lures the murderer into this | trap |
| 478 | 30 | d | 2 | No CS | Der Kommissar lockt den Mörder | in diese | Falle | The commissioner lures the murderer into this | trap |
| 479 | 30 | d | 3 | Semantic | Der Kommissar lockt den Mörder | in diese | Spüle | The commissioner lures the murderer into this | sink |
| 480 | 30 | d | 4 | CS at N | Der Kommissar lockt den Mörder | in diese | lovušku | The commissioner lures the murderer into this | trap |
| 481 | 31 | a | 1 | No CS | Der Sohn räumt das Geschirr | in diese | Spüle | The son removes the dishes into this | sink |
| 482 | 31 | a | 2 | Semantic | Der Sohn räumt das Geschirr | in diese | Ader | The son removes the dishes into this | vein |
| 483 | 31 | a | 3 | CS at N | Der Sohn räumt das Geschirr | in diese | mojku | The son removes the dishes into this | sink |
| 484 | 31 | a | 4 | CS at P | Der Sohn räumt das Geschirr | v ėtu | mojku | The son removes the dishes into this | sink |
| 485 | 31 | b | 1 | Semantic | Der Enkel räumt das Besteck | in diese | Ader | The grandson removes the cutlery into this | vein |
| 486 | 31 | b | 2 | CS at N | Der Enkel räumt das Besteck | in diese | mojku | The grandson removes the cutlery into this | sink |
| 487 | 31 | b | 3 | CS at P | Der Enkel räumt das Besteck | v ėtu | mojku | The grandson removes the cutlery into this | sink |
| 488 | 31 | b | 4 | No CS | Der Enkel räumt das Besteck | in diese | Spüle | The grandson removes the cutlery into this | sink |
| 489 | 31 | c | 1 | CS at N | Die Küchenhilfe räumt die Teller | in diese | mojku | The kitchen help removes the plates into this | sink |
| 490 | 31 | c | 2 | CS at P | Die Küchenhilfe räumt die Teller | v ėtu | mojku | The kitchen help removes the plates into this | sink |
| 491 | 31 | c | 3 | No CS | Die Küchenhilfe räumt die Teller | in diese | Spüle | The kitchen help removes the plates into this | sink |
| 492 | 31 | c | 4 | Semantic | Die Küchenhilfe räumt die Teller | in diese | Ader | The kitchen help removes the plates into this | vein |
| 493 | 31 | d | 1 | CS at P | Der Koch räumt die Gläser | v ėtu | mojku | The cook removes the glasses into this | sink |
| 494 | 31 | d | 2 | No CS | Der Koch räumt die Gläser | in diese | Spüle | The cook removes the glasses into this | sink |
| 495 | 31 | d | 3 | Semantic | Der Koch räumt die Gläser | in diese | Ader | The cook removes the glasses into this | vein |
| 496 | 31 | d | 4 | CS at N | Der Koch räumt die Gläser | in diese | mojku | The cook removes the glasses into this | sink |
| 497 | 32 | a | 1 | No CS | Der Arzthelfer spritzt das Medikament | in diese | Ader | The doctor's assistant injects the medication into this | vein |
| 498 | 32 | a | 2 | Semantic | Der Arzthelfer spritzt das Medikament | in diese | Falle | The doctor's assistant injects the medication into this | trap |
| 499 | 32 | a | 3 | CS at N | Der Arzthelfer spritzt das Medikament | in diese | venu | The doctor's assistant injects the medication into this | vein |
| 500 | 32 | a | 4 | CS at P | Der Arzthelfer spritzt das Medikament | v ėtu | venu | The doctor's assistant injects the medication into this | vein |
| 501 | 32 | b | 1 | Semantic | Die Krankenschwester spritzt den Impfstoff | in diese | Falle | The nurse injects the vaccine into this | trap |
| 502 | 32 | b | 2 | CS at N | Die Krankenschwester spritzt den Impfstoff | in diese | venu | The nurse injects the vaccine into this | vein |
| 503 | 32 | b | 3 | CS at P | Die Krankenschwester spritzt den Impfstoff | v ėtu | venu | The nurse injects the vaccine into this | vein |
| 504 | 32 | b | 4 | No CS | Die Krankenschwester spritzt den Impfstoff | in diese | Ader | The nurse injects the vaccine into this | vein |
| 505 | 32 | c | 1 | CS at N | Der Krankenpfleger spritzt das Heilmittel | in diese | venu | The nurse injects the medicament into this | vein |
| 506 | 32 | c | 2 | CS at P | Der Krankenpfleger spritzt das Heilmittel | v ėtu | venu | The nurse injects the medicament into this | vein |
| 507 | 32 | c | 3 | No CS | Der Krankenpfleger spritzt das Heilmittel | in diese | Ader | The nurse injects the medicament into this | vein |
| 508 | 32 | c | 4 | Semantic | Der Krankenpfleger spritzt das Heilmittel | in diese | Falle | The nurse injects the medicament into this | trap |
| 509 | 32 | d | 1 | CS at P | Der Doktor spritzt die Flüssigkeit | v ėtu | venu | The doctor injects the liquid into this | vein |
| 510 | 32 | d | 2 | No CS | Der Doktor spritzt die Flüssigkeit | in diese | Ader | The doctor injects the liquid into this | vein |
| 511 | 32 | d | 3 | Semantic | Der Doktor spritzt die Flüssigkeit | in diese | Falle | The doctor injects the liquid into this | trap |
| 512 | 32 | d | 4 | CS at N | Der Doktor spritzt die Flüssigkeit | in diese | venu | The doctor injects the liquid into this | vein |
| 513 | 33 | a | 1 | No CS | Der Reporter schreibt die Notiz | auf diesen | Zettel | The reporter writes the note onto this | slip of paper |
| 514 | 33 | a | 2 | Semantic | Der Reporter schreibt die Notiz | auf diesen | Keller | The reporter writes the note onto this | cellar |
| 515 | 33 | a | 3 | CS at N | Der Reporter schreibt die Notiz | auf diesen | listok | The reporter writes the note onto this | slip of paper |
| 516 | 33 | a | 4 | CS at P | Der Reporter schreibt die Notiz | na ėtot | listok | The reporter writes the note onto this | slip of paper |
| 517 | 33 | b | 1 | Semantic | Der Schüler schreibt den Satz | auf diesen | Keller | The pupil writes the sentence onto this | cellar |
| 518 | 33 | b | 2 | CS at N | Der Schüler schreibt den Satz | auf diesen | listok | The pupil writes the sentence onto this | slip of paper |
| 519 | 33 | b | 3 | CS at P | Der Schüler schreibt den Satz | na ėtot | listok | The pupil writes the sentence onto this | slip of paper |
| 520 | 33 | b | 4 | No CS | Der Schüler schreibt den Satz | auf diesen | Zettel | The pupil writes the sentence onto this | slip of paper |
| 521 | 33 | c | 1 | CS at N | Die Assistentin schreibt die Telefonnummer | auf diesen | listok | The assistent writes the telephone number onto this | slip of paper |
| 522 | 33 | c | 2 | CS at P | Die Assistentin schreibt die Telefonnummer | na ėtot | listok | The assistent writes the telephone number onto this | slip of paper |
| 523 | 33 | c | 3 | No CS | Die Assistentin schreibt die Telefonnummer | auf diesen | Zettel | The assistent writes the telephone number onto this | slip of paper |
| 524 | 33 | c | 4 | Semantic | Die Assistentin schreibt die Telefonnummer | auf diesen | Keller | The assistent writes the telephone number onto this | cellar |
| 525 | 33 | d | 1 | CS at P | Der Mitarbeiter schreibt die Adresse | na ėtot | listok | The colleague writes the address onto this | slip of paper |
| 526 | 33 | d | 2 | No CS | Der Mitarbeiter schreibt die Adresse | auf diesen | Zettel | The colleague writes the address onto this | slip of paper |
| 527 | 33 | d | 3 | Semantic | Der Mitarbeiter schreibt die Adresse | auf diesen | Keller | The colleague writes the address onto this | cellar |
| 528 | 33 | d | 4 | CS at N | Der Mitarbeiter schreibt die Adresse | auf diesen | listok | The colleague writes the address onto this | slip of paper |
| 529 | 34 | a | 1 | No CS | Der Schneider hängt den Anzug | in diesen | Schrank | The tailor hangs the suit into this | wardrobe |
| 530 | 34 | a | 2 | Semantic | Der Schneider hängt den Anzug | in diesen | Wald | The tailor hangs the suit into this | forest |
| 531 | 34 | a | 3 | CS at N | Der Schneider hängt den Anzug | in diesen | škaf | The tailor hangs the suit into this | wardrobe |
| 532 | 34 | a | 4 | CS at P | Der Schneider hängt den Anzug | v ėtot | škaf | The tailor hangs the suit into this | wardrobe |
| 533 | 34 | b | 1 | Semantic | Der Diener hängt das Kleid | in diesen | Wald | The servant hangs the dress into this | forest |
| 534 | 34 | b | 2 | CS at N | Der Diener hängt das Kleid | in diesen | Schrank | The servant hangs the dress into this | wardrobe |
| 535 | 34 | b | 3 | CS at P | Der Diener hängt das Kleid | v ėtot | škaf | The servant hangs the dress into this | wardrobe |
| 536 | 34 | b | 4 | No CS | Der Diener hängt das Kleid | in diesen | Schrank | The servant hangs the dress into this | wardrobe |
| 537 | 34 | c | 1 | CS at N | Der Butler hängt den Mantel | in diesen | škaf | The butler hangs the coat into this | wardrobe |
| 538 | 34 | c | 2 | CS at P | Der Butler hängt den Mantel | v ėtot | škaf | The butler hangs the coat into this | wardrobe |
| 539 | 34 | c | 3 | No CS | Der Butler hängt den Mantel | in diesen | Schrank | The butler hangs the coat into this | wardrobe |
| 540 | 34 | c | 4 | Semantic | Der Butler hängt den Mantel | in diesen | Wald | The butler hangs the coat into this | forest |
| 541 | 34 | d | 1 | CS at P | Die Schülerin hängt den Pullover | v ėtot | škaf | The pupil hangs the sweater into this | wardrobe |
| 542 | 34 | d | 2 | No CS | Die Schülerin hängt den Pullover | in diesen | Schrank | The pupil hangs the sweater into this | wardrobe |
| 543 | 34 | d | 3 | Semantic | Die Schülerin hängt den Pullover | in diesen | Wald | The pupil hangs the sweater into this | forest |
| 544 | 34 | d | 4 | CS at N | Die Schülerin hängt den Pullover | in diesen | škaf | The pupil hangs the sweater into this | wardrobe |
| 545 | 35 | a | 1 | No CS | Der Arzt legt das Pflaster | auf diese | Wunde | The doctor puts the band-aid onto this | wound |
| 546 | 35 | a | 2 | Semantic | Der Arzt legt das Pflaster | auf diese | Tasse | The doctor puts the band-aid onto this | cup |
| 547 | 35 | a | 3 | CS at N | Der Arzt legt das Pflaster | auf diese | ranu | The doctor puts the band-aid onto this | wound |
| 548 | 35 | a | 4 | CS at P | Der Arzt legt das Pflaster | na ėtu | ranu | The doctor puts the band-aid onto this | wound |
| 549 | 35 | b | 1 | Semantic | Der Sanitäter legt die Bandage | auf diese | Tasse | The paramedic puts the bandage onto this | cup |
| 550 | 35 | b | 2 | CS at N | Der Sanitäter legt die Bandage | auf diese | ranu | The paramedic puts the bandage onto this | wound |
| 551 | 35 | b | 3 | CS at P | Der Sanitäter legt die Bandage | na ėtu | ranu | The paramedic puts the bandage onto this | wound |
| 552 | 35 | b | 4 | No CS | Der Sanitäter legt die Bandage | auf diese | Wunde | The paramedic puts the bandage onto this | wound |
| 553 | 35 | c | 1 | CS at N | Die Krankenschwester legt den Verband | auf diese | ranu | The nurse puts the brace onto this | wound |
| 554 | 35 | c | 2 | CS at P | Die Krankenschwester legt den Verband | na ėtu | ranu | The nurse puts the brace onto this | wound |
| 555 | 35 | c | 3 | No CS | Die Krankenschwester legt den Verband | auf diese | Wunde | The nurse puts the brace onto this | wound |
| 556 | 35 | c | 4 | Semantic | Die Krankenschwester legt den Verband | auf diese | Tasse | The nurse puts the brace onto this | cup |
| 557 | 35 | d | 1 | CS at P | Der Krankenpfleger legt das Heftpflaster | na ėtu | ranu | The nurse puts the band-aid onto this | wound |
| 558 | 35 | d | 2 | No CS | Der Krankenpfleger legt das Heftpflaster | auf diese | Wunde | The nurse puts the band-aid onto this | wound |
| 559 | 35 | d | 3 | Semantic | Der Krankenpfleger legt das Heftpflaster | auf diese | Tasse | The nurse puts the band-aid onto this | cup |
| 560 | 35 | d | 4 | CS at N | Der Krankenpfleger legt das Heftpflaster | auf diese | ranu | The nurse puts the band-aid onto this | wound |
| 561 | 36 | a | 1 | No CS | Der Bäcker streut den Zucker | auf diesen | Kuchen | The baker sprinkles the sugar onto this | cake |
| 562 | 36 | a | 2 | Semantic | Der Bäcker streut den Zucker | auf diesen | Schuppen | The baker sprinkles the sugar onto this | shed |
| 563 | 36 | a | 3 | CS at N | Der Bäcker streut den Zucker | auf diesen | pirog | The baker sprinkles the sugar onto this | cake |
| 564 | 36 | a | 4 | CS at P | Der Bäcker streut den Zucker | na ėtot | pirog | The baker sprinkles the sugar onto this | cake |
| 565 | 36 | b | 1 | Semantic | Der Konditor streut die Nüsse | auf diesen | Schuppen | The pastry chef sprinkles the nuts onto this | shed |
| 566 | 36 | b | 2 | CS at N | Der Konditor streut die Nüsse | auf diesen | pirog | The pastry chef sprinkles the nuts onto this | cake |
| 567 | 36 | b | 3 | CS at P | Der Konditor streut die Nüsse | na ėtot | pirog | The pastry chef sprinkles the nuts onto this | cake |
| 568 | 36 | b | 4 | No CS | Der Konditor streut die Nüsse | auf diesen | Kuchen | The pastry chef sprinkles the nuts onto this | cake |
| 569 | 36 | c | 1 | CS at N | Die Bäckersfrau streut den Puderzucker | auf diesen | pirog | The baker sprinkles the powdered sugar onto this | cake |
| 570 | 36 | c | 2 | CS at P | Die Bäckersfrau streut den Puderzucker | na ėtot | pirog | The baker sprinkles the powdered sugar onto this | cake |
| 571 | 36 | c | 3 | No CS | Die Bäckersfrau streut den Puderzucker | auf diesen | Kuchen | The baker sprinkles the powdered sugar onto this | cake |
| 572 | 36 | c | 4 | Semantic | Die Bäckersfrau streut den Puderzucker | auf diesen | Schuppen | The baker sprinkles the powdered sugar onto this | shed |
| 573 | 36 | d | 1 | CS at P | Der Zuckerbäcker streut die Mandeln | na ėtot | pirog | The confectioner sprinkles the almonds onto this | cake |
| 574 | 36 | d | 2 | No CS | Der Zuckerbäcker streut die Mandeln | auf diesen | Kuchen | The confectioner sprinkles the almonds onto this | cake |
| 575 | 36 | d | 3 | Semantic | Der Zuckerbäcker streut die Mandeln | auf diesen | Schuppen | The confectioner sprinkles the almonds onto this | shed |
| 576 | 36 | d | 4 | CS at N | Der Zuckerbäcker streut die Mandeln | auf diesen | pirog | The confectioner sprinkles the almonds onto this | cake |
| 577 | 37 | a | 1 | No CS | Die Nachbarin schüttet das Abwasser | in diesen | Abfluss | The neighbour pours the wastewater into this | drain |
| 578 | 37 | a | 2 | Semantic | Die Nachbarin schüttet das Abwasser | in diesen | Zettel | The neighbour pours the wastewater into this | slip of paper |
| 579 | 37 | a | 3 | CS at N | Die Nachbarin schüttet das Abwasser | in diesen | sliv | The neighbour pours the wastewater into this | drain |
| 580 | 37 | a | 4 | CS at P | Die Nachbarin schüttet das Abwasser | v ėtot | sliv | The neighbour pours the wastewater into this | drain |
| 581 | 37 | b | 1 | Semantic | Der Klempner schüttet das Schmutzwasser | in diesen | Zettel | The plumber pours the dirty water into this | slip of paper |
| 582 | 37 | b | 2 | CS at N | Der Klempner schüttet das Schmutzwasser | in diesen | sliv | The plumber pours the dirty water into this | drain |
| 583 | 37 | b | 3 | CS at P | Der Klempner schüttet das Schmutzwasser | v ėtot | sliv | The plumber pours the dirty water into this | drain |
| 584 | 37 | b | 4 | No CS | Der Klempner schüttet das Schmutzwasser | in diesen | Abfluss | The plumber pours the dirty water into this | drain |
| 585 | 37 | c | 1 | CS at N | Der Handwerker schüttet das Spülwasser | in diesen | sliv | The craftsman pours the dishwater into this | drain |
| 586 | 37 | c | 2 | CS at P | Der Handwerker schüttet das Spülwasser | v ėtot | sliv | The craftsman pours the dishwater into this | drain |
| 587 | 37 | c | 3 | No CS | Der Handwerker schüttet das Spülwasser | in diesen | Abfluss | The craftsman pours the dishwater into this | drain |
| 588 | 37 | c | 4 | Semantic | Der Handwerker schüttet das Spülwasser | in diesen | Zettel | The craftsman pours the dishwater into this | slip of paper |
| 589 | 37 | d | 1 | CS at P | Der Mechaniker schüttet das Öl | v ėtot | sliv | The mechanic pours the oil into this | drain |
| 590 | 37 | d | 2 | No CS | Der Mechaniker schüttet das Öl | in diesen | Abfluss | The mechanic pours the oil into this | drain |
| 591 | 37 | d | 3 | Semantic | Der Mechaniker schüttet das Öl | in diesen | Zettel | The mechanic pours the oil into this | slip of paper |
| 592 | 37 | d | 4 | CS at N | Der Mechaniker schüttet das Öl | in diesen | sliv | The mechanic pours the oil into this | drain |
| 593 | 38 | a | 1 | No CS | Der Junge schleppt die Kartons | in diesen | Keller | The boy carries the cardboard boxes into this | cellar |
| 594 | 38 | a | 2 | Semantic | Der Junge schleppt die Kartons | in diesen | Umschlag | The boy carries the cardboard boxes into this | envelope |
| 595 | 38 | a | 3 | CS at N | Der Junge schleppt die Kartons | in diesen | podval | The boy carries the cardboard boxes into this | cellar |
| 596 | 38 | a | 4 | CS at P | Der Junge schleppt die Kartons | v ėtot | podval | The boy carries the cardboard boxes into this | cellar |
| 597 | 38 | b | 1 | Semantic | Der Freund schleppt die Kisten | in diesen | Umschlag | The friend carries the boxes into this | envelope |
| 598 | 38 | b | 2 | CS at N | Der Freund schleppt die Kisten | in diesen | podval | The friend carries the boxes into this | cellar |
| 599 | 38 | b | 3 | CS at P | Der Freund schleppt die Kisten | v ėtot | podval | The friend carries the boxes into this | cellar |
| 600 | 38 | b | 4 | No CS | Der Freund schleppt die Kisten | in diesen | Keller | The friend carries the boxes into this | cellar |
| 601 | 38 | c | 1 | CS at N | Der Holzfäller schleppt das Brennholz | in diesen | podval | The lumberjack carries the firewood into this | cellar |
| 602 | 38 | c | 2 | CS at P | Der Holzfäller schleppt das Brennholz | v ėtot | podval | The lumberjack carries the firewood into this | cellar |
| 603 | 38 | c | 3 | No CS | Der Holzfäller schleppt das Brennholz | in diesen | Keller | The lumberjack carries the firewood into this | cellar |
| 604 | 38 | c | 4 | Semantic | Der Holzfäller schleppt das Brennholz | in diesen | Umschlag | The lumberjack carries the firewood into this | envelope |
| 605 | 38 | d | 1 | CS at P | Der Student schleppt die Vorräte | v ėtot | podval | The student carries the provisions into this | cellar |
| 606 | 38 | d | 2 | No CS | Der Student schleppt die Vorräte | in diesen | Keller | The student carries the provisions into this | cellar |
| 607 | 38 | d | 3 | Semantic | Der Student schleppt die Vorräte | in diesen | Umschlag | The student carries the provisions into this | envelope |
| 608 | 38 | d | 4 | CS at N | Der Student schleppt die Vorräte | in diesen | podval | The student carries the provisions into this | cellar |
| 609 | 39 | a | 1 | No CS | Der Umzugshelfer lädt die Möbel | auf diesen | Lastwagen | The removal man loads the furniture onto this | truck |
| 610 | 39 | a | 2 | Semantic | Der Umzugshelfer lädt die Möbel | auf diesen | Briefkasten | The removal man loads the furniture onto this | letter box |
| 611 | 39 | a | 3 | CS at N | Der Umzugshelfer lädt die Möbel | auf diesen | gruzovik | The removal man loads the furniture onto this | truck |
| 612 | 39 | a | 4 | CS at P | Der Umzugshelfer lädt die Möbel | na ėtot | gruzovik | The removal man loads the furniture onto this | truck |
| 613 | 39 | b | 1 | Semantic | Der Arbeiter lädt die Waren | auf diesen | Briefkasten | The worker loads the goods onto this | letter box |
| 614 | 39 | b | 2 | CS at N | Der Arbeiter lädt die Waren | auf diesen | gruzovik | The worker loads the goods onto this | truck |
| 615 | 39 | b | 3 | CS at P | Der Arbeiter lädt die Waren | na ėtot | gruzovik | The worker loads the goods onto this | truck |
| 616 | 39 | b | 4 | No CS | Der Arbeiter lädt die Waren | auf diesen | Lastwagen | The worker loads the goods onto this | truck |
| 617 | 39 | c | 1 | CS at N | Der Spediteur lädt die Kartons | auf diesen | gruzovik | The haulage contractor loads the boxes onto this | truck |
| 618 | 39 | c | 2 | CS at P | Der Spediteur lädt die Kartons | na ėtot | gruzovik | The haulage contractor loads the boxes onto this | truck |
| 619 | 39 | c | 3 | No CS | Der Spediteur lädt die Kartons | auf diesen | Lastwagen | The haulage contractor loads the boxes onto this | truck |
| 620 | 39 | c | 4 | Semantic | Der Spediteur lädt die Kartons | auf diesen | Briefkasten | The haulage contractor loads the boxes onto this | letter box |
| 621 | 39 | d | 1 | CS at P | Der Möbelpacker lädt die Tische | na ėtot | gruzovik | The mover loads the tables onto this | truck |
| 622 | 39 | d | 2 | No CS | Der Möbelpacker lädt die Tische | auf diesen | Lastwagen | The mover loads the tables onto this | truck |
| 623 | 39 | d | 3 | Semantic | Der Möbelpacker lädt die Tische | auf diesen | Briefkasten | The mover loads the tables onto this | letter box |
| 624 | 39 | d | 4 | CS at N | Der Möbelpacker lädt die Tische | auf diesen | gruzovik | The mover loads the tables onto this | truck |
| 625 | 40 | a | 1 | No CS | Die Sanitäter legen die Verletzten | auf diese | Tragen | The paramedics lay the injured persons onto these | stretchers |
| 626 | 40 | a | 2 | Semantic | Die Sanitäter legen die Verletzten | auf diese | Eimer | The paramedics lay the injured persons onto these | buckets |
| 627 | 40 | a | 3 | CS at N | Die Sanitäter legen die Verletzten | auf diese | nosilki | The paramedics lay the injured persons onto these | stretchers |
| 628 | 40 | a | 4 | CS at P | Die Sanitäter legen die Verletzten | na ėti | nosilki | The paramedics lay the injured persons onto these | stretchers |
| 629 | 40 | b | 1 | Semantic | Die Ärzte legen die Kranken | auf diese | Eimer | The doctors lay the sick persons onto these | buckets |
| 630 | 40 | b | 2 | CS at N | Die Ärzte legen die Kranken | auf diese | nosilki | The doctors lay the sick persons onto these | stretchers |
| 631 | 40 | b | 3 | CS at P | Die Ärzte legen die Kranken | na ėti | nosilki | The doctors lay the sick persons onto these | stretchers |
| 632 | 40 | b | 4 | No CS | Die Ärzte legen die Kranken | auf diese | Tragen | The doctors lay the sick persons onto these | stretchers |
| 633 | 40 | c | 1 | CS at N | Die Pfleger legen die Patienten | auf diese | nosilki | The nurses lay the patients onto these | stretchers |
| 634 | 40 | c | 2 | CS at P | Die Pfleger legen die Patienten | na ėti | nosilki | The nurses lay the patients onto these | stretchers |
| 635 | 40 | c | 3 | No CS | Die Pfleger legen die Patienten | auf diese | Tragen | The nurses lay the patients onto these | stretchers |
| 636 | 40 | c | 4 | Semantic | Die Pfleger legen die Patienten | auf diese | Eimer | The nurses lay the patients onto these | buckets |
| 637 | 40 | d | 1 | CS at P | Die Krankenschwestern legen die Verwundeten | na ėti | nosilki | The nurses lay the wounded persons onto these | stretchers |
| 638 | 40 | d | 2 | No CS | Die Krankenschwestern legen die Verwundeten | auf diese | Tragen | The nurses lay the wounded persons onto these | stretchers |
| 639 | 40 | d | 3 | Semantic | Die Krankenschwestern legen die Verwundeten | auf diese | Eimer | The nurses lay the wounded persons onto these | buckets |
| 640 | 40 | d | 4 | CS at N | Die Krankenschwestern legen die Verwundeten | auf diese | nosilki | The nurses lay the wounded persons onto these | stretchers |
